# Supplementary material for: Eggs for Improving Nutrition, cognitive development and reducing linear growth retardation among Infants and young Children (ENRICH): protocol of an egg supplementation trial among children aged 9–18 months in Hyderabad, India
Source: BMJ Paediatr Open. 2024 Feb 27;8(Suppl 1):e001892. doi: 10.1136/bmjpo-2023-001892 (PMC10900354; doi:10.1136/bmjpo-2023-001892)
Supplement: Supplementary data [file bmjpo-2023-001892supp001.pdf]

Appendix 1: Questionnaires for data collection

1. Egg preparation and delivery

2) Date and time of the cooking (Date and time)

3) When it was delivered (Time)

4) Did the child receive the egg with in the time (4 h from cooking time)?

   a) Yes

   b) No

4) If no, what is the reason

   1. Traffic
   2. The mother/ family members were not available at home
   3. The mother/ family members rejected the egg
   96. Other specify

2. Daily Compliance questionnaire

1 Did the child receive the egg yesterday

   1. Yes
   2. No

2 If yes, did the child consume the egg yesterday

   1. Yes
   2. No

3 Which portion of the egg is given to the child?

   1. Only egg white
   2. Only egg yolk/yellow
   3. Whole egg

4 At what time the child consumed the egg

   Date and time (recorded)

6 If No, why was it not consumed yesterday

   1. The child disliked the taste
   2. The child fell sick/illness/diarrhoea/vomiting
   3. Family members/others advised not to give
   96. Other, specify

3. Monthly follow-up questionnaire (Control)

1. Are you getting eggs from AWC: yes/No
2. If yes how many eggs did you receive last month?
   a. 16 b. >16 c.<16
3. Have you started egg feeding: Yes/No
4. If Yes, how frequently eggs were consumed in the previous week
   a. Everyday b. 4-6 days c. 1-3 days d. Never
5. What is the portion size usually eaten by the child
   a. >1 egg. B. 1 egg. C. ½ egg. D. ¼ egg
6. Was the egg mixed with other food for consumption: Yes/ No
7. Number of times egg eaten per day
   a. Once. B. 2 times. C. 3 times. D. More than 4 times
8. Which portion of the egg was readily accepted by the child:
   a. Yolk. B. White. C. Whole D. None
9. Did your child experience any of the following difficulties after egg consumption
   a. Fever. B. Cough. C. Vomitings& Diarrhoea. D. Wheezing. E. tooth ache F None
10. Have you noticed any change in the child's appetite
    a. Yes, increased. B. Yes, Decreased. C. No change
11. In the previous week have you fed eggs procured from any other sources:yes/No
12. If yes, mention the source__________

4. Pre-intervention questionnaire (knowledge and practice), (in intervention and control at endline; only in intervention at the beginning and at midline)

PART 1: PRACTICE

1. Do you currently breastfeed your child?
   a. Yes
   b. No

2. How many times do you breastfeed the child in a day? (Duration of breastfeeding should be at least 10 minutes)
   a. Less than 4 times
   b. 4 times
   c. 5-6 times
   d. More than 6 times

3. Do you give complementary food to your child? [Dropdown]
   a. Yes
   b. No

4. At what age did you initiate complementary feeding to your child?
   a. Less than 6 months
   b. 6 months
   c. 7 months
   d. 8 months
   e. Not yet started,
   f. Other, specify_________

5. If not yet started, what is the reason for not starting complementary feeding to your child?
   a. Child is too small to eat solid foods
   b. Child doesn't have teeth to chew food
   c. Elders advised not to
   d. Fear of indigestion

6. What are the types of complementary foods that are currently given to the child? [Multiple options can be selected]
   a. Not yet started
   b. Formula milk (Eg: Lactogen,amul milk spray etc . . .)
   c. Commercial baby foods (Eg: Ceralac, farex,nestem etc. . .)
   d. Homemade semi-solids (Eg: Uggu, rice+ dhal (soft cooked), mash fruits&vegetables etc. . .)
   e. Processed food (Eg:Biscuits,kurkure,chips etc . . .)

Banjara SK*, et al. BMJ Paediatrics Open* 2024; 8:e001892. doi: 10.1136/bmjpo-2023-001892

f. Homemade solids (Eg: Normal food)
g. Other, specify_______

7. What are the foods that are generally included in the homemade complementary foods? (logic)[Multiple options can be selected]
   a. Cereals & millets
   b. Pulses
   c. Green leafy vegetables
   d. Roots & tubers
   e. Other vegetables
   f. Fruits
   g. Flesh foods
   h. Eggs
   i. Milk & milk products
   j. Fat & oils
   k. Sugar & jaggery.

8. Do you feed eggs to your child? Skip Logic (Questions regarding feeding egg, only if the mother is feeding egg to her child)
   a. Yes
   b. No

9. If yes, at what age did you start feeding eggs to your child?
   a. 6 months
   b. 7 months
   c. 8 months
   d. 9 months
   e. Other, specify _________

10. If not, what is the reason for not initiating egg to the child before 9 months?
   a. The eggs do not get digested
   b. The eggs cause heat build-up in the child's body
   c. Family members suggested to avoid eggs till 8th month
   d. Eggs are high in fat/cholesterol
   e. Other, specify________________

11. Who suggested not to initiate egg to your child?
   a. Husband
   b. Mother-in-law
   c. Neighbours
   d. Doctor
   e. Other, specify ____________

12. What form of the egg did your child like to eat?
   a. Boiled Egg
   b. Half boiled
   c. Omelette /Scrambled Egg
   d. Egg Curry
   e. Mashed with rice
   f. Other, specify ____________

13. Did you stop feeding eggs to your child after initiating?
    a. Yes
    b. No

14. If yes, why did you stop feeding eggs to your child?
    a. The child disliked the taste
    b. The child had an allergy
    c. The child had diarrhoea/vomiting
    d. Family members/others advised not to give.
    e. Other, specify__________________

15. If the child did not like the taste, what did you do?
    a. Tried a different recipe with egg
    b. Stopped feeding egg
    c. Other, specify_____________

16. If the child developed an allergy/diarrhoea/ vomiting after consuming the egg, whom did you contact?
    a. Government hospital / PHC doctor
    b. Private doctor/Registered Medical Practitioner
    c. Local medical shop
    d. Traditional/ Religious leaders
    e. Treated at home
    f. Family members/neighbours suggested
    g. Other, specify ____________

17. Does any member of the family have an allergy to any food items?
    a. Yes
    b. No

18. Who has an allergy to the food item?
    a. Father of the child
    b. Mother of the child
    c. Grandparents of the child
    d. Siblings

19. Which food is allergic to the above-mentioned person?
    ____

20. How do you feed eggs to your child? [MCQ- Multiple options can be selected]
    a. Mashed with rice
    b. Boiled Egg
    c. Half boiled
    d. Omelette /Scrambled Egg
    e. Egg Curry
    f. Other, specify _________________

21. Which part of the egg is given to the child? [Drop down]

a. Only egg white
b. Only egg yolk
c. Whole egg

22. Why do you give only the white part of the egg to the child? [If selected a]-
a. Child likes the taste
b. Easy to swallow
c. Family members / elders advised
d. Doctor advised
e. ASHA/ ANM/Anganwadi worker advised.
f. Other, specify__________________

23. Why do you give only the yellow part to the child? [If selected b]
a. Child likes the taste
b. Easy to swallow
c. Family members/elders advised
d. Doctor advised
e. ASHA/ ANM/Anganwadi worker advised.
f. Other, specify __________________

24. How frequently do you feed eggs to your child? [Drop down]
a. Daily
b. Twice a week
c. Thrice a week
d. >4 times a week
e. Weekly once

25. Usually, how many eggs do you feed your child in a day? [MCQ]
a. One whole egg
b. Half egg
c. Two whole eggs
d. One whole egg white
e. Two whole egg white
f. One whole egg yolk
g. Two whole egg yolk
h. Other, specify____________________

26. If the egg is not fed -, please tell us the reason from the options provided below [Multiple options can be selected]
a. Not available and accessible
b. The child dislikes eating egg
c. No time to cook the egg
d. Family members do not prefer
e. Other, specify___________________

27. Do you avoid feeding eggs to your child on festivals/special days in a week? [Skip Logic-If yes, go to Q. 21, If not, go to 23]
a. Yes
b. No

28. If yes, please mention on which days in the week do you avoid feeding eggs to the child? [Short answer]

____

29. If yes, please mention on which occasions do you avoid feeding eggs to the child? (Prompt: Festivals, seasons) [Short answer]

____

Banjara SK, *et al. BMJ Paediatrics Open* 2024; 8:e001892. doi: 10.1136/bmjpo-2023-001892

PART 2: KNOWLEDGE

This section is to know the knowledge of the mother regarding feeding egg to the child

30. Are eggs an important component of the child's diet for their growth and development [Drop down]
    a. Yes
    b. No
    c. Don't know

31. What are the benefits of feeding an egg to the child? [Multiple options can be selected]
    a. Eggs are good for vision
    b. Eggs help the child grow tall
    c. Eggs provide immunity
    d. Eggs are good for bones and teeth
    e. Eggs increase brain capacity
    f. All of the above
    g. None of the above
    h. I don't know
    i. Other, specify________

32. Which part of the egg is healthy for the child? [Drop down]
    a. Only Egg yolk
    b. Only Egg white
    c. Whole egg
    d. Don't know

33. At what age the children should start consuming eggs? [Drop down]

    ____

34. I intend to feed one whole egg daily to the child from 9 months of age [Drop down]
    a. Extremely unlikely
    b. Unlikely
    c. Neutral
    d. Likely
    e. Extremely likely

35. I am confident that I can feed one whole egg to the child every day from 9 months of age [Drop down]
    a. Not confident
    b. Confident
    c. Very confident
    d. Neutral

36. Husband and elders in the family will support in feeding egg to the child from 9 monthsof age [Drop down]

a. Agree
b. Disagree
c. Don't know

37. Would you like to know about the recipes you can prepare and feed your child? [Dropdown]
a. Yes
b. No

ICDS

38. Did you enrol your or your child's name in the local ICDS center/ Anganwadi? [SkipLogic- If not, go to 44]
a. Yes
b. No

38. a If yes, What are the benefits you are getting from the ICDS centre (Multiple choices can be selected)
a. Arogya Lakshmi one full meal (During pregnancy and lactation of 6 months)
b. Balamrutham
c. Eggs
d. Iron and folic tablets
e. Milk
f. Others, please specify__________________________

39. How many eggs do you receive from the ICDS center in a month?
a. 16
b. Less than 16
c. Don't know /remember

40. Have you received less than 16 eggs anytime from the ICDS center/ Anganwadi?
a. Yes
b. No

41. If yes, do you know the reason for receiving less than 16 eggs from the ICDS center?
a. No
b. Yes, specify

42. How frequently are the eggs given from the ICDS center are consumed by your child in the last 30 days? [Skip Logic-Skip next question, if selected Option. a, Go to next question if selected option. b./c./d.]
a. Often
b. Rarely
c. Never consumed

43. If the number of eggs fed is less than the allowance, what is the reason? [MCQ]
a. Not received or received less number of eggs from the center
b. The child is too young to eat the egg
c. Shared it with family members
d. Discarded
e. Other, specify ______

44. Is there any organization/non-governmental organization (NGO) that sponsors eggs for your family?
    a. Yes
    b. No

45. If yes, what is the name of the organization? (Short answer)

____

46. What is the number of eggs that you receive from the organization mentioned above?

____

5. Preliminary screening (For both intervention and control)

1. Will you relocate to a new place in the next 9 months period?

a.Yes

b.No

c.Maybe

2.Do you give complementary food to your child?

a.Yes

b.No

3.Do you consume eggs at your home?

a. Yes

b.No

4.Have you started feeding eggs to your child?

a. Yes

b.No

5.Does your child have allergic reactions after consumption of egg?

a.Yes

b.No

c. Don't know

6.Have any other allergic reactions to any other food item occurred to your child?

a. Yes

b. No

c. Don't know

7. Which food item is your child allergic to?

____

8. Is any of the family member allergic to any food items?

a. Yes

b. No

c. Maybe

9. Which family member has allergic reactions to food item?

a. Mother b. Father c. Siblings

d. Grandparents.

10. Which food item does the above-mentioned person has allergic reactions to?

____

11. Does your child have any of the below mentioned conditions?

a. Congenital anomalies/diseases

b. Chronic morbidity

c. None

12. Will you be willing to initiate egg consumption for your child and be a part of this egg supplementation group if randomly selected from 9 to 18 months of your child's age?

a. Yes

b. No

13. Did your child get MMR vaccination?

a. Yes

b. No

Appendix 2

Protocol for Management of egg allergy

Since egg allergies typically present immediately or within two hours after ingestion, an auxiliary nurse midwife will be present during the first egg feed and she will observe the child for any for signs of egg allergies or anaphylaxis for a period of 2 hours. In case of allergy/anaphylaxis, allergies will be clinically evaluated by a paediatrician and treated clinically with a referral to tertiary hospital for management after giving a dose of antihistamine in the field unit of the study.

Second generation antihistamines and the dose will be kept with the Staff nurse for use in case of emergency.

-Cetirizine 6 months to < 2 years: 0.25 mg/kg;

- Levocetirizine 6 months to 5 years: 1.25 mg OD

Any child exhibiting egg allergy will be excluded from the study.

Banjara SK, *et al. BMJ Paediatrics Open* 2024; 8:e001892. doi: 10.1136/bmjpo-2023-001892
