# Supplementary material for: Eggs for Improving Nutrition, cognitive development and reducing linear growth retardation among Infants and young Children (ENRICH): protocol of an egg supplementation trial among children aged 9–18 months in Hyderabad, India
Source: BMJ Paediatr Open. 2024 Feb 27;8(Suppl 1):e001892. doi: 10.1136/bmjpo-2023-001892 (PMC10900354; doi:10.1136/bmjpo-2023-001892)
Supplement: Author's manuscript [file bmjpo-2023-001892.draft_revisions.pdf]

BMJ Paediatrics Open

BMJ Paediatrics Open is committed to open peer review. As part of this commitment we make the peer review history of every article we publish publicly available.

When an article is published we post the peer reviewers' comments and the authors' responses online. We also post the versions of the paper that were used during peer review. These are the versions that the peer review comments apply to.

The versions of the paper that follow are the versions that were submitted during the peer review process. They are not the versions of record or the final published versions. They should not be cited or distributed as the published version of this manuscript.

BMJ Paediatrics Open is an open access journal and the full, final, typeset and author-corrected version of record of the manuscript is available on our site with no access controls, subscription charges or pay-per-view fees (http://bmjpaedsopen.bmj.com).

If you have any questions on BMJ Paediatrics Open's open peer review process please email info.bmjpo@bmj.com

BMJ Paediatrics Open

Eggs for Improving Nutrition, cognitive development and Reducing linear growth retardation among Infants and young Children (ENRICH): protocol of an egg supplementation trial among 9-18 months old children in Hyderabad, India

Journal:	*BMJ Paediatrics Open*
Manuscript ID	bmjpo-2023-001892
Article Type:	Protocol
Date Submitted by the Author:	04-Feb-2023

Complete List of Authors:	B, Santosh Kumar; National Institute of Nutrition, Clinical Division<br>Challa, Sai; National Institute of Nutrition, Maternal and Child Health, Nutrition<br>Augustine, Little Flower; ICMR-National Institute of Nutrition<br>Dasi, Teena; National Institute of Nutrition<br>Madhari, Radhika; ICMR - National Institute of Nutrition, Hyderabad, India<br>Fernandez Rao, Sylvia; National Institute of Nutrition<br>Palika, Ravindranadh; National Institute of Nutrition, Drug Safety Division; NATIONAL INSTITUTE OF NUTRITION<br>Pullakhandam, Raghu; National Institute of Nutrition<br>Kalashikam, Rajender Rao; National Institute of Nutrition<br>Kumar, Ramachandrappa Naveen; National Institute of Nutrition<br>Palepu, Dharani; National Institute of Nutrition, Clinical Division<br>Chilumula, Monica; National Institute of Nutrition, Maternal and Child Health, Nutrition<br>Selvaraj, Kiruthika; National Institute of Nutrition<br>Gopinath, Preethi; National Institute of Nutrition<br>Davies-Kershaw, Hilary; LSHTM, Department of Population Health; London School of Hygiene and Tropical Medicine<br>Jobarteh, Modou; London School of Hygiene & Tropical Medicine, Epidemiology & Population Health<br>Haggarty, Paul; University of Aberdeen<br>Ferguson, Elaine; London School of Hygiene and Tropical Medicine (LSHTM)<br>Heffernan, Claire; University of London, Department of Pathobiology and Population Sciences; London International Development Centre (LIDC)<br>Kulkarni, Bharati; National Institute of Nutrition

Keywords:	Nutrition, Epidemiology, Growth, Child Psychiatry, Health services research

I, the Submitting Author has the right to grant and does grant on behalf of all authors of the Work (as defined in the below author licence), an exclusive licence and/or a non-exclusive licence for contributions from authors who are: i) UK Crown employees; ii) where BMJ has agreed a CC-BY licence shall apply, and/or iii) in accordance with the terms applicable for US Federal Government officers or employees acting as part of their official duties; on a worldwide, perpetual, irrevocable, royalty-free basis to BMJ Publishing Group Ltd ("BMJ") its licensees and where the relevant Journal is co-owned by BMJ to the co-owners of the Journal, to publish the Work in this journal and any other BMJ products and to exploit all rights, as set out in our licence.

The Submitting Author accepts and understands that any supply made under these terms is made by BMJ to the Submitting Author unless you are acting as an employee on behalf of your employer or a postgraduate student of an affiliated institution which is paying any applicable article publishing charge ("APC") for Open Access articles. Where the Submitting Author wishes to make the Work available on an Open Access basis (and intends to pay the relevant APC), the terms of reuse of such Open Access shall be governed by a Creative Commons licence – details of these licences and which Creative Commons licence will apply to this Work are set out in our licence referred to above.

Other than as permitted in any relevant BMJ Author's Self Archiving Policies, I confirm this Work has not been accepted for publication elsewhere, is not being considered for publication elsewhere and does not duplicate material already published. I confirm all authors consent to publication of this Work and authorise the granting of this licence.

Confidential: For Review Only

Eggs for Improving Nutrition, cognitive development and Reducing linear growth retardation among Infants and young Children (ENRICH): protocol of an egg supplementation trial among 9-18 months old children in Hyderabad, India

Santosh Kumar Banjara, Sai Ram Challa, Little Flower Augustine, Teena Dasi, Radhika Madhari, Sylvia Fernandez Rao, Ravindranadh Palika, Raghu Pullakhandam, Rajender Rao Kalashikam, Naveen Kumar Ramachandrappa, Dharani Prathyusha, Monica Chilumula, Kiruthika Selvaraj, Preethi Anigula, Hilary Davies-Kershaw, Modou Jobarteh, Paul Haggarty, Claire Heffernan, Elaine Ferguson, Bharati Kulkarni

Corresponding author: Sai Ram Challa, MBBS, MD

Scientist 'E', Head, Clinical Division (Maternal& Child Health and Nutrition), ICMR-National Institute of Nutrition, Hyderabad, India

Email: drsairamnin@gmail.com

Phone: +91-9618774603

Confidential: For Review Only

Abstract

Introduction

Evidence on the impact of nutrient-rich animal source foods like eggs for improving child growth and cognition is inconsistent. This study aims to examine the impact of an egg intervention in children, along with behaviour change communication (BCC) to the mother, on linear growth and cognition, and nutritional status in 9-18 month old children.

Methods and analysis

A 9-month open-labelled randomized controlled trial will be conducted in 3 urban slums in Hyderabad, India, as a sub-study of an observational cohort study (n=350) following pregnant women and their children until 18 months of age in a population at risk of stunting. The children born to women enrolled during the third trimester of pregnancy will be block randomized in a 1:4 ratio into the intervention (n=70) and control (n= 280) groups. Children in the intervention group will be supplemented with one egg per day starting from 9 months until 18 months of age. BCC designed to enhance adherence to the intervention will be used. The control group will be a part of the observational cohort and will not receive any intervention from the study team. The primary outcome will be length-for-age z-scores, and the secondary outcomes will include cognition, blood biomarkers of nutritional status including fatty acid profile, and epigenetic signatures linked with linear growth and cognition. Multivariate intention-to-treat analyses will be conducted to assess the effect of the intervention.

Ethics and dissemination

The study is approved by the Institutional ethics committees of ICMR-National Institute of Nutrition, Hyderabad, India and London School of Hygiene and Tropical Medicine, UK. The results will be published in peer-reviewed journals and disseminated to policymakers. Findings will also be shared with study participants and community leaders.

Trial Registration number: CTRI/2021/11/038208

Confidential: For Review Only

Key messages

What is known about the subject

- Previous studies using single or multiple micronutrients or cereal-legume based food supplements have shown only modest impact on child stunting.
- Eggs provide several critical nutrients to support the infant growth and development and egg supplementation is a promising option for nutrition programmes.
- Evidence on the impact of nutrient dense animal source foods like eggs for improving child growth and cognition is inconsistent.

What this study hopes to add

- Evidence on the impact of daily egg supplementation during 9 to 18 months of age on the linear growth, cognition, nutritional biomarkers and epigenetic outcomes
- Information on intrauterine and early infancy growth in participants would allow evaluation of the potential of egg intervention as a modifier of linear growth trajectory

For Review Only

Introduction

The high prevalence of stunting in children under five years of age in India (35%)[1] remains a cause of concern given the associated long-term negative consequences on cognition, physical work capacity, earning potential, and risk of chronic diseases in adulthood[2 3]. Childhood stunting is common among populations residing in adverse environments with limited access to nutrient-dense foods and poor sanitation[4]. In these environments, children's diets are predominantly cereal-based, with low amounts of animal source foods (ASFs) that contain high quality complete protein, and do not meet the high nutrient needs of young children[5]. Improving the quality of early childhood diets and reducing child stunting is therefore a public health priority in India.

Previous evidence based on randomized controlled trials (RCTs) evaluating supplementation with cereal legume-based complementary foods or single or multiple micronutrient interventions in children have shown a limited impact on linear growth[6-8]. The emerging evidence suggests that locally available ASFs like eggs could play an important role in improving linear growth of young children[9]. However, the evidence is mixed, and the impact of an egg intervention on linear growth and stunting prevalence appears to vary in different settings depending on the dietary patterns and baseline prevalence of child stunting. For example, an RCT in 163 children (6–9 months) from Ecuador assessed the impact of egg supplementation (1 egg per day for 6 months) and showed a reduction in the prevalence of stunting by 47% and underweight by 74% in the intervention arm (n=83)[10]. However, in a similar trial in 660 children (6-9 months) from Malawi, an egg intervention did not have a beneficial effect on linear growth, possibly due to low baseline prevalence of stunting and background diets being rich in ASFs[11]. In another RCT from Malawi (9 month old children, N=267), the addition of egg powder with bovine colostrum to complementary feeds resulted in lower prevalence of stunting in the intervention arm (n=135, RR = 0.70; 95% CI: 0.52, 0.94)[12]. The aetiology of stunting is complex and poorly understood and the effect of interventions may differ between countries and ethnicities. There is no evidence available from the Indian subcontinent, where the stunting prevalence is high, and the ASF intake is low.

https://mc.manuscriptcentral.com/bmjpo

Confidential: For Review Only

Eggs provide several nutrients critical to infant growth and development which include all the essential amino acids making it a high-quality/complete protein, docosahexaenoic acid (DHA), and micronutrients such as vitamin B12 which are important for neural development and immune function[9]. Eggs are among the richest sources of choline, which is necessary for the production of phospholipids, cell membrane integrity, and the conversion of acetylcholine and sphingomyelin necessary for brain development and function[13]. Dietary choline intake can modulate DNA methylation, therefore, egg supplementation may also impact the epigenetic signatures[14]. Epigenetics is one of the candidate mechanisms linking early life factors to physical and cognitive development with consequences across the life course[15-18]. Evaluation of epigenetics signatures linked with stunting is central to the Action Against Stunting Hub (AASH) observational cohort[19].

In India, eggs are more affordable than other ASFs and are relatively simple to store and prepare. Egg supplementation is a promising option in government nutrition programs. In recent years, a few states in India have included eggs in supplementary feeding programs for pregnant women and children in the age group 7 months to 6 years[20 21]. However, systematic impact evaluation of these interventions on anthropometric, biochemical, or cognitive outcomes has not been done. We hypothesize that daily egg supplementation (accompanied by behaviour change communication (BCC) to increase compliance to the intervention) starting at 9 months will improve linear growth and reduce the incidence of stunting at 18 months of age.

The proposed ENRICH study aims to evaluate the effect of daily supplementation with an egg in children from 9 months to 18 months of age (accompanied by behaviour change advice to the mother to increase compliance with the intervention) on:

1) linear growth and stunting at 18 months
2) cognition, nutritional status, and relevant epigenetic signatures.

Confidential: For Review Only

Methods and analysis

Study design and setting

The ENRICH study is a two-arm individually randomized open-labelled controlled trial nested within the AASH cohort study [22] (Figure 1). The study will be conducted in three urban slums (Addagutta, Warasiguda and Sitaphalmandi) in Hyderabad, India. These slums altogether have a population of ≈175,000 and previous studies in this area have found a high prevalence of childhood stunting (around 27% at 24months age)[8].Women will be recruited in the study in the third trimester of pregnancy, and the egg intervention will start when the children are 9 months old. Data and sample collection will be conducted as per the observational study protocol, including infant blood samples at 6 and 18 months; anthropometric measurements, dietary data and infant cognitive function at 9, 12 and 18 months of age. Blinding the participants is not possible due to the nature of the intervention, but the study staff measuring study outcomes will be blinded to the intervention group[22].

Sample size

Assuming α = 0.05 and 90% power and a difference of 0.32 in the length-for-age z score (LAZ) between the intervention and control groups with standard deviation (SD)= 0.70 and an allocation ratio of 1:4 in the intervention and control arms, accounting for a 10% loss to follow up, the estimated sample size is 69 children (rounded off to 70) in the intervention group and 277 (rounded off to 280) children in the control arm.

The estimated effect size is comparable to the other trials on complementary feeding interventions and is a conservative estimate of half the effect size in a similar study carried out in Ecuador[10]. For comparison of blood biomarkers relevant for the egg intervention, a random sub-sample of 70 children in the control group will be selected. This sample size is adequate to detect a difference of 0.35 µg/mL in choline as observed in the above study in Ecuador with about 90% power.

Confidential: For Review Only

Outcome measures

The primary study outcome is the LAZ score at 18 months. The secondary outcomes include cognition (INTER-NDA component of the INTERGROWTH), plasma concentrations of insulin-like growth factor 1(IGF-1), insulin-like growth factor binding protein-3 (IGFBP3), serum/plasma concentrations of essential amino acids, micronutrient status including; retinol, folate, vitamin B12, betaine, as well as red blood cell (RBC) fatty acids, DHA, choline and epigenetic (DNA methylation) signatures linked to the study outcomes. The detailed methodology has been described elsewhere[22].

Participant recruitment

The present trial is nested within the AASH observational cohort study where pregnant women (n=700) in the third trimester of pregnancy residing in the urban slums are being enrolled through the primary health centres (PHC) after eligibility assessment and obtaining written informed consent. The data collection in pregnancy and during the follow up of the offspring has been described elsewhere[22]. Of these, 350 children born from February 2022 to March 2023 will be a part of the present trial; 70 children will be randomized to the intervention arm and 280 to the control arm. The control arm children will also be a part of the observational cohort.

Eligibility

The inclusion criteria during pregnancy will be: healthy pregnant women in the age group 18-40 y, gestation 26-32 weeks, singleton pregnancy and willingness to comply with the study requirements. Exclusion criteria for children include congenital anomalies that could impact on key study outcomes; known egg allergy, allergic reaction to egg during test feeding or history of anaphylaxis or severe allergic reaction to any substance.

Randomization, intervention allocation and blinding

Each month, all children between 7.5 and 8.5 months of age will be randomly assigned to either the intervention or control group in a ratio of 1:4 after ascertaining the eligibility and

Confidential: For Review Only

obtaining written informed consent from the mother. Project staff collecting data on study outcomes will be blinded to the allocation sequence.

Through a centralised block randomization system, 350 children will be randomly allocated to blocks of 5 or 10 children, maintaining a ratio of 1:4 intervention and control children within each block. Allocation is concealed until randomisation.

Trial intervention, intervention delivery, and quality control

Egg supplementation will start on completion of 9 months of child age. One hen's egg (medium-sized, weighing around 50 g, raw / boiled depending on the mother's choice) will be delivered to children's homes every day by project staff, and mothers will be asked to feed it to the child within four hours. The mothers will be provided instructions on age-appropriate egg-based complementary food recipes through a purpose-made video. The importance of feeding the egg to the index child and not sharing it with other family members will also be stressed in this video to improve adherence to the intervention. This is a model of BCC developed based on evidence generated through best practices for Take – home rations of supplementary nutrition by Government of India[23]. The specific messages were developed through formative research conducted with parents and grandmothers of young children. The project staff will observe the first feeding episode of complementary food containing eggs, and mothers will be counselled in case of minor gastric problems. Study staff will visit the households twice a week to observe the child feeding in the first month of the intervention and will encourage and support the mothers. Information on consumption of an egg by the child on the previous day is collected daily by the field staff while delivering the eggs in the intervention group. For comparison, data on eggs consumption will also be collected every month during a home visit in the control group children.

The eggs will be procured locally from a verified supplier. The quality control measures to ensure food safety of the intervention will include periodic assessment of the microbial contamination of eggs: both at the point of purchase and the point of use. Random sub-samples of eggs collected soon after purchase (6 eggs each month) and complementary food prepared using eggs for the child (from 6 households every month) will be analysed for

https://mc.manuscriptcentral.com/bmjpo

Confidential: For Review Only

microbial contamination (Salmonella, Shigella, total coliform count) at the NIN laboratory. The nutrient composition of the eggs will be assessed for major nutrients (energy, proteins, iron, folate, calcium, vitamin A, D etc) every three months at the food chemistry laboratory of ICMR- NIN.

Control group

The state government currently provides eggs (16 eggs per month for 7 months to 3 years) for beneficiaries of Integrated child development services (ICDS, a Government initiative for all women and children who can voluntarily enrol) as a part of the Rations. However, during formative research with mothers(n=100), it was found that children below one year of age are not fed whole eggs due to fear of indigestion and other reasons. Moreover, 59% of children were not fed eggs supplied through the government's supplementary nutrition programs due to intra-household sharing[24] . Therefore, our intervention will include a strong BCC focusing on the nutritional benefits of feeding whole eggs to young children. There will not be any active intervention by the study team in the control group. Nevertheless, the 'contamination' of BCC messages in the control group is possible in this individually randomized trial. Moreover, all children from both the intervention and control groups may receive eggs as a part of Take-Home Ration, which may reduce the magnitude of the observed effect of the intervention. The number of eggs received through Government programme and the number of eggs consumed by the child will be recorded for the intervention as well as control group. Therefore, we will assess the mother's knowledge of the nutritional benefits of eggs in both the groups at baseline and 18 months for comparison between the two groups.

Data collection at baseline and during follow up

Data collection in both the intervention and control groups would be done following the same protocol as the observational cohort. The parameters to be measured at different time points are listed in Table 1. Details on the methodology for assessing primary and secondary outcomes are described elsewhere[22]. A trained phlebotomist will draw a 3 ml venous blood sample at both six months and 18 months in children from the intervention

Confidential: For Review Only

group and a sub-sample of 70 children from the control groups. The methods for assessment of blood parameters and epigenetic signatures linked with stunting and cognition have been described elsewhere[19 22]. Data on morbidity (including information on episodes of hospital admission and sick-child clinic visits) will be collected using a structured questionnaire every two weeks. Mothers will be encouraged to report any adverse events to the study staff. Field data will be collected using the CommCare software application in electronic tablets.

Withdrawal from the study

During the consent process, mothers will be informed that they may choose to withdraw their child from the study at any time. Children whose mothers wish to withdraw after the randomization will be visited by a project staff to discuss and record the reasons for withdrawal.

In case of suggestive of egg allergy symptoms, the participants would be referred to the PHC, and the mother would be advised to consult the medical officer and seek advice on the continuation of egg supplementation. Data on the prevalence of egg allergy in Indian children is not available but based on the self-reported prevalence of egg allergy (< 2.5%) in other settings[25], around 1-2 cases are anticipated.

If a participant withdraws from the trial, data captured will contribute to the analysis up to the day prior to discontinuation, unless the mother wishes to withdraw consent for any data captured up to that point. Participants who withdraw from the study will not be replaced.

Statistical analysis

We will summarise demographic, clinical, and laboratory data using descriptive statistics. The primary analysis will be based on the intention to treat (ITT) population and will compare the children's LAZ scores at the age of 18 months in the intervention and control groups using generalized linear regression models. We will adjust the models for key

Confidential: For Review Only

covariates (E.g., age, gender and area) associated with the outcome variables. We will check the model assumptions and make appropriate adjustments to the analysis where necessary. We will also use difference-in-difference analysis to evaluate the impact of the intervention. We will compare the secondary outcome measures between the two groups using ITT principles. The study is powered for detecting meaningful differences in linear growth and DHA concentrations in the two groups. Other secondary outcome analyses will be exploratory. Additional analysis of the primary and secondary endpoints will also be presented using the per protocol population. No interim analysis is planned[26 27].

Patient and Public Involvement

Patients were not directly involved in the design or conduct of the study. Formative research involving mothers, grandmothers and others from the local community (excluding study participants) assisted in understanding local cultural practices and developing BCC material [24].

Ethics and dissemination

This protocol, informed consent documents, and patient information sheets have been reviewed and approved by the Research Ethics Committees at ICMR-NIN (CR/3/IV/2022 dated 28.7.2022) and LSHTM (IRB no. 18029) as well as by an independent Data Safety Monitoring Board (DSMB). The trial is registered with the Clinical Trials Registry of India (CTRI/2021/11/038208). The DSMB is independent from the sponsor and competing interests and will review clinical data at regular intervals as the study progresses.
The study results will be disseminated to the local and national government departments (for potential modifications in the supplementary nutrition program); academic channels like national conferences and published in peer-reviewed scientific journals.

Confidential: For Review Only

Significance and impact

The objectives of the present trial are well aligned with the objectives of the National Nutrition Mission in India, with the potential for immediate scale-up of the intervention. The study has several strengths. Chief among them is the comprehensive evaluation of outcome indicators ranging from linear growth, cognition, nutritional biomarkers and epigenetic states indicative of exposures and biological function. Additional information on maternal diets and blood biomarkers in pregnancy as well as information on the intrauterine growth of the offspring would allow us to examine pathways for the impact that previous studies have not reported. A limitation is that in this community-based intervention, egg consumption by the index child cannot be supervised completely. Moreover, '''contamination' of the BCC messages in the control group and contamination related to the Government's supplementary nutrition programme may attenuate the effect size. Nevertheless, if the trial demonstrates significant benefits, the evidence on the impact of this relatively inexpensive yet nutrient rich ASF in early life will inform strategies for stunting reduction in India and other developing countries.

Funding

The study is funded by the UK Research and Innovation, Global Challenges Research Fund.

'Authors' contributions:

SKB, SRC, BK: Conceptualized, designed the study and wrote first draft

LA, TD, DP, MC, KS, PA: developed detailed protocols, planned data collection, critical input to manuscript

RM, HD, CH, SFR, RNP, RP, RRK, NKR, MJ, PH: concept, design, critical inputs to manuscript

Confidential: For Review Only

References

1. Comprehensive National Nutrition Survey (CNNS) National Report. Population Council: Ministry of health and family welfare, Government of India, UNICEF, 2018.
2. Hoffman DJ, Sawaya AL, Verreschi I, et al. Why are nutritionally stunted children at increased risk of obesity? Studies of metabolic rate and fat oxidation in shantytown children from Sao Paulo, Brazil. *The American journal of clinical nutrition* 2000;72(3):702-07.
3. Martins VJ, Toledo Florêncio TM, Grillo LP, et al. Long-lasting effects of undernutrition. *International journal of environmental research and public health* 2011;8(6):1817-46.
4. World Health Organization. Reducing stunting in children: equity considerations for achieving the global targets 2025. Geneva: World Health Organization, 2018.
5. Kulkarni B. Addressing the double burden of malnutrition in developing countries: Need for strategies to improve the lean body mass. *Food and nutrition bulletin* 2018;39(2_suppl):S69-S76.
6. Christian P, Shaikh S, Shamim AA, et al. Effect of fortified complementary food supplementation on child growth in rural Bangladesh: a cluster-randomized trial. *International journal of epidemiology* 2015;44(6):1862-76.
7. Lassi ZS, Das JK, Zahid G, et al. Impact of education and provision of complementary feeding on growth and morbidity in children less than 2 years of age in developing countries: a systematic review. *BMC public health* 2013;13(3):1-10.
8. Radhakrishna K, Hemalatha R, Geddam JB, et al. Effectiveness of zinc supplementation to full term normal infants: a community based double blind, randomized, controlled, clinical trial. *PloS one* 2013;8(5):e61486.
9. Lutter CK, Iannotti LL, Stewart CP. The potential of a simple egg to improve maternal and child nutrition. *Maternal & child nutrition* 2018;14:e12678.
10. Iannotti LL, Lutter CK, Stewart CP, et al. Eggs in early complementary feeding and child growth: a randomized controlled trial. *Pediatrics* 2017;140(1)
11. Stewart CP, Caswell B, Iannotti L, et al. The effect of eggs on early child growth in rural Malawi: the Mazira Project randomized controlled trial. *The American journal of clinical nutrition* 2019;110(4):1026-33.
12. Bierut T, Duckworth L, Grabowsky M, et al. The effect of bovine colostrum/egg supplementation compared with corn/soy flour in young Malawian children: A randomized, controlled clinical trial. *The American Journal of Clinical Nutrition* 2021;113(2):420-27.
13. Iannotti L, Lutter C, Bunn D, et al. Eggs: The uncracked potential for improving maternal and young child nutrition among the world's. *Issue: Nutrition Reviews, 72 (6)* 2014
14. Zeisel SH. Choline, other methyl-donors and epigenetics. *Nutrients* 2017;9(5):445.
15. Tiffon C. The impact of nutrition and environmental epigenetics on human health and disease. *International journal of molecular sciences* 2018;19(11):3425.
16. Haggarty P, Ferguson-Smith AC, Kuh D, et al. Life course epigenetics and healthy ageing. *A life course approach to healthy ageing* 2013:198-212.
17. Lorgen-Ritchie M, Murray AD, Staff R, et al. Imprinting methylation predicts hippocampal volumes and hyperintensities and the change with age in later life. *Scientific reports* 2021;11(1):1-12.
18. Lorgen-Ritchie M, Murray AD, Ferguson-Smith AC, et al. Imprinting methylation in SNRPN and MEST1 in adult blood predicts cognitive ability. *Plos one* 2019;14(2):e0211799.

https://mc.manuscriptcentral.com/bmjpo

19. Anouschka R, Magatte N, Rajender RaoKalashikam et al. Epigenetic Studies in Families and Children at Risk of Stunting in India, Indonesia, and Senegal. BMJ pediatrics Open, 2022.
20. Kulkarni B, Mamidi R, Balakrishna N, et al. Body composition assessment in infancy and early childhood: comparison of anthropometry with dual-energy X-ray absorptiometry in low-income group children from India. *European journal of clinical nutrition* 2014;68(6):658-63.
21. Department of women and child development. Government of Telangana, 2022.
22. Davies-Kershaw H, Fahmida U, Kyaw HM, et al. Anthropometric, biochemical, dietary, morbidity and wellbeing assessments in women and children in Indonesia, India and Senegal: a UKRI GCRF Action Against Stunting Hub protocol paper. BMJ Pediatrics Open, 2022.
23. NITI Aayog and World food programme. Take home ration: Good practices across the States/UTs: NITI Aayog, 2022.
24. Thomas N, Kulkarni B, Augustine L, et al. Formative Study to Identify the Determinants, Current Practices and Knowledge of Mothers related to the Egg Consumption of Young Children in Hyderabad, Telangana. Abstract No. OP-2022-0098. 54th Annual conference of Nutrition Society of India. Hyderabad: ICMR-National Institute of Nutrition, Hyderabad, 2022.
25. Muraro A, Halken S, Arshad S, et al. EAACI food allergy and anaphylaxis guidelines. Primary prevention of food allergy. *Allergy* 2014;69(5):590-601.
26. McCoy CE. Understanding the Intention-to-treat Principle in Randomized Controlled Trials. *The western journal of emergency medicine* 2017;18(6):1075-78. doi: 10.5811/westjem.2017.8.35985 [published Online First: 2017/11/01]
27. Rosenblum M. Robust Analysis of RCTs Using Generalized Linear Models. Targeted Learning: Springer 2011:187-99.

Confidential: For Review Only

TABLE 1. Data collection during the follow up period

Measure	**Mother**	**Infant** (intervention and control group)
Buccal/saliva sample for epigenetics	At enrolment	At birth and at 18 months
• Multiple pass24-hour diet recall with repeat in 10% of the sample<br>• Initiation of breast feeding<br>• Exclusive breastfeeding and introduction to complementary foods,<br>• Multiple pass 24-hour dietary recall with repeat in 10% of the sample	In 3rd trimester of pregnancy	Breast feeding questionnaire at birth<br>Breastfeeding questionnaire at 1-6 months<br><br>Dietary 24-hour recall at 6, 9, 12 and 18 months
Micronutrient content of the breastmilk samples: vitamin A, B12, folate	At 3 months postpartum	
Anthropometry measurements<br>Standing and sitting height, weight, skinfolds and MUAC<br><br>Length, weight, Head circumference, MUAC, knee-heel length and skinfolds	In 3rd trimester of pregnancy<br>Weight only at 1, 2, 4 and 5 months after birth	At birth and at 3,6,9,12, 18 and 24 months<br>Weight only at 1, 2, 4 and 5 months.

Confidential: For Review Only

Micronutrient status: Hemoglobin, serum ferritin, serum transferrin receptors, growth hormone,serum iron, zinc, retinol, folate, vitamin B12, folate, IGF1, IGFBP3	In 3rd trimester of pregnancy	At 6 and 18 months
Blood biomarkers relevant for egg intervention:RBC fatty acids, amino acids, choline, betaine		At 9 and 18 months (in the intervention group and in a sub-sample of 69 children from control group)
Inflammatory markers in blood:CRP and AGP		At 6 and 18 months
Stool for entero-pathogens, parasites, and inflammatory markers MPO, AAT		At 6 and 18 months
Assessment of cognition with context specific tests		Home Observation Measurement of the Environment (HOME -Scale): at 10-14 monthsand18 months<br>Oxford Neurodevelopment Assessment (OX-NDA): at 10-14 months<br>INTER- Neurodevelopment assessment 9INTER-NDA): At 22-24 months
Assessment of morbidity using structured pictorial charts to be completed daily		Completed by mother each week from birth until 18 months everytwo weeks throughout the follow up
Assessment of home environment		Using a structured questionnaire at 9 months

Confidential: For Review Only

Figure 1: CONSORT Flowchart of the study design

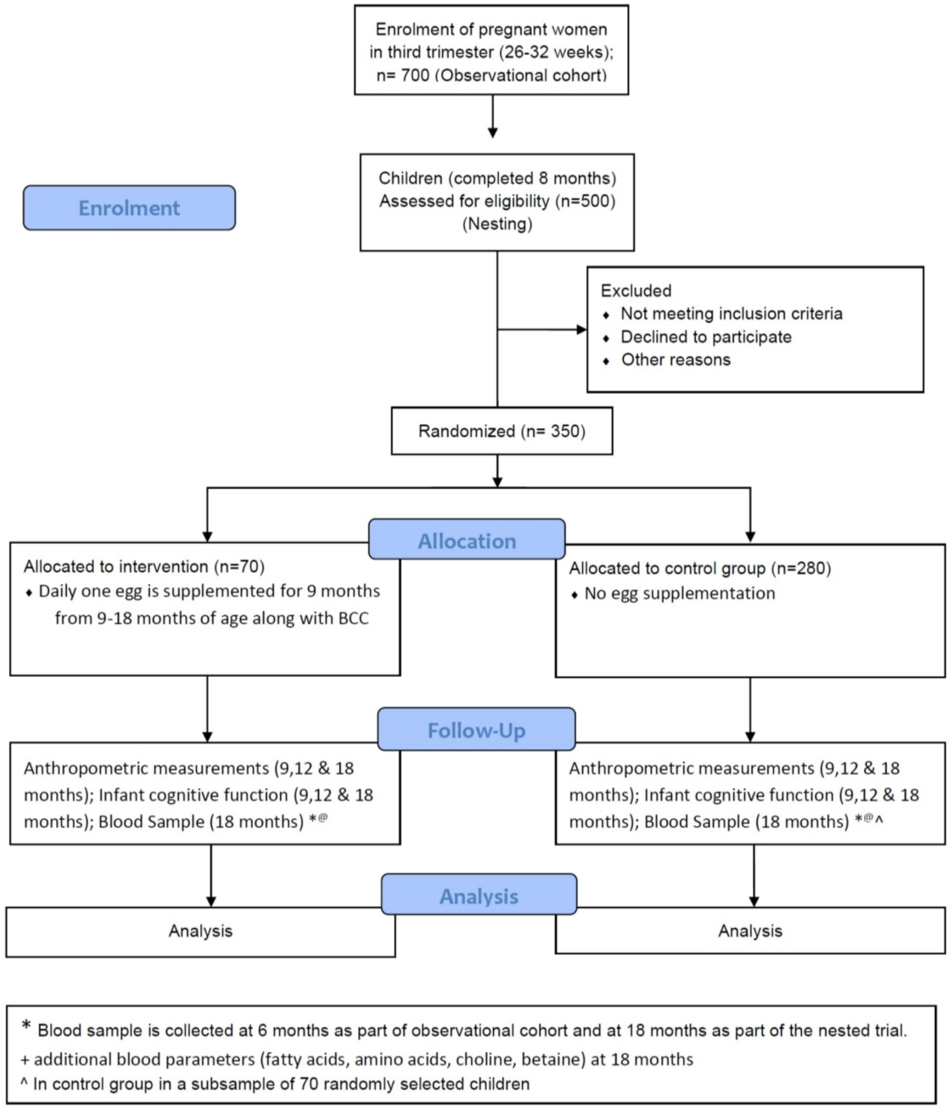

Figure 1: CONSORT Flowchart of the study design

176x214mm (300 x 300 DPI)

BMJ Paediatrics Open

Eggs for Improving Nutrition, cognitive development and Reducing linear growth retardation among Infants and young Children (ENRICH): protocol of an egg supplementation trial among 9-18 months old children in Hyderabad, India

Journal:	*BMJ Paediatrics Open*
Manuscript ID	bmjpo-2023-001892.R1
Article Type:	Protocol
Date Submitted by the Author:	16-May-2023

Complete List of Authors:	Banjara, Santosh Kumar; National Institute of Nutrition, Clinical Division<br>Challa, Sai Ram; National Institute of Nutrition, Maternal and Child Health, Nutrition<br>Augustine, Little Flower; National Institute of Nutrition<br>Dasi, Teena; National Institute of Nutrition<br>Madhari, Radhika; ICMR - National Institute of Nutrition, Hyderabad, India<br>Fernandez Rao, Sylvia; National Institute of Nutrition<br>Palika, Ravindranadh; National Institute of Nutrition, Drug Safety Division; NATIONAL INSTITUTE OF NUTRITION<br>Pullakhandam, Raghu; National Institute of Nutrition<br>Kalashikam, Rajender Rao; National Institute of Nutrition<br>Kumar, Ramachandrappa Naveen; National Institute of Nutrition<br>Palepu, Dharani; National Institute of Nutrition, Clinical Division<br>Chilumula, Monica; National Institute of Nutrition, Maternal and Child Health, Nutrition<br>Selvaraj, Kiruthika; National Institute of Nutrition<br>Gopinath, Preethi; National Institute of Nutrition<br>Davies-Kershaw, Hilary; LSHTM, Department of Population Health; London School of Hygiene and Tropical Medicine<br>Jobarteh, Modou; London School of Hygiene & Tropical Medicine, Epidemiology & Population Health<br>Haggarty, Paul; University of Aberdeen<br>Ferguson, Elaine; London School of Hygiene and Tropical Medicine (LSHTM)<br>Heffernan, Claire; University of London, Department of Pathobiology and Population Sciences; London International Development Centre (LIDC)<br>Kulkarni, Bharati; National Institute of Nutrition

Keywords:	Nutrition, Epidemiology, Growth, Child Psychiatry, Health services research

I, the Submitting Author has the right to grant and does grant on behalf of all authors of the Work (as defined in the below author licence), an exclusive licence and/or a non-exclusive licence for contributions from authors who are: i) UK Crown employees; ii) where BMJ has agreed a CC-BY licence shall apply, and/or iii) in accordance with the terms applicable for US Federal Government officers or employees acting as part of their official duties; on a worldwide, perpetual, irrevocable, royalty-free basis to BMJ Publishing Group Ltd ("BMJ") its licensees and where the relevant Journal is co-owned by BMJ to the co-owners of the Journal, to publish the Work in this journal and any other BMJ products and to exploit all rights, as set out in our licence.

The Submitting Author accepts and understands that any supply made under these terms is made by BMJ to the Submitting Author unless you are acting as an employee on behalf of your employer or a postgraduate student of an affiliated institution which is paying any applicable article publishing charge ("APC") for Open Access articles. Where the Submitting Author wishes to make the Work available on an Open Access basis (and intends to pay the relevant APC), the terms of reuse of such Open Access shall be governed by a Creative Commons licence – details of these licences and which Creative Commons licence will apply to this Work are set out in our licence referred to above.

Other than as permitted in any relevant BMJ Author's Self Archiving Policies, I confirm this Work has not been accepted for publication elsewhere, is not being considered for publication elsewhere and does not duplicate material already published. I confirm all authors consent to publication of this Work and authorise the granting of this licence.

Confidential: For Review Only

Eggs for Improving Nutrition, cognitive development and Reducing linear growth retardation among Infants and young Children (ENRICH): protocol of an egg supplementation trial among 9-18 months old children in Hyderabad, India

Santosh Kumar Banjara, Sai Ram Challa, Little Flower Augustine, Teena Dasi, Radhika Madhari, Sylvia Fernandez Rao, Ravindranadh Palika, Raghu Pullakhandam, Rajender Rao Kalashikam, Naveen Kumar Ramachandrappa, Dharani Prathyusha, Monica Chilumula, Kiruthika Selvaraj, Preethi Anigula, Hilary Davies-Kershaw, Modou Jobarteh, Paul Haggarty, Claire Heffernan, Elaine Ferguson, Bharati Kulkarni

Corresponding author: Sai Ram Challa, MBBS, MD

Scientist 'E', Head, Clinical Division (Maternal& Child Health and Nutrition), ICMR-National Institute of Nutrition, Hyderabad, India

Email: drsairamnin@gmail.com

Phone: +91-9618774603

Confidential: For Review Only

Abstract

Introduction

Evidence on the impact of nutrient-rich animal source foods like eggs for improving child growth and cognition is inconsistent. This study aims to examine the impact of an egg intervention in children, along with behaviour change communication (BCC) to the mother, on linear growth and cognition, and nutritional status in 9-18 month old children.

Methods and analysis

A 9-month open-labelled randomized controlled trial will be conducted in 3 urban slums in Hyderabad, India, as a sub-study of an observational cohort study (n=350) following pregnant women and their children until 18 months of age in a population at risk of stunting. The children born to women enrolled during the third trimester of pregnancy will be block randomized in a 1:4 ratio into the intervention (n=70) and control (n= 280) groups. Children in the intervention group will be supplemented with one egg per day starting from 9 months until 18 months of age. BCC designed to enhance adherence to the intervention will be used. The control group will be a part of the observational cohort and will not receive any intervention from the study team. The primary outcome will be length-for-age z-scores, and the secondary outcomes will include cognition, blood biomarkers of nutritional status including fatty acid profile, and epigenetic signatures linked with linear growth and cognition. Multivariate intention-to-treat analyses will be conducted to assess the effect of the intervention.

Ethics and dissemination

The study is approved by the Institutional ethics committees of ICMR-National Institute of Nutrition, Hyderabad, India and London School of Hygiene and Tropical Medicine, UK. The results will be published in peer-reviewed journals and disseminated to policymakers. Findings will also be shared with study participants and community leaders.

Trial Registration number: CTRI/2021/11/038208

Confidential: For Review Only

Key messages

What is known about the subject

- Previous studies using single or multiple micronutrients or cereal-legume-based food supplements have shown a modest impact on child stunting.
- Evidence on the impact of nutrient-dense animal source foods like eggs for improving child growth and cognition is inconsistent.

What this study hopes to add

- Comprehensive evidence on the impact of daily egg supplementation during 9 to 18 months of age on linear growth, cognition, nutritional biomarkers, and epigenetic outcomes
-

How this study might affect research, practice, or policy

- Eggs provide several critical nutrients to support infant growth and development; egg supplementation is a promising option for nutrition programs.
- Therefore, the trial can potentially scale up to National programmes.

For Review Only

Introduction

The high prevalence of stunting in children under five years of age in India (35%)[1] remains a cause of concern given the associated long-term negative consequences on cognition, physical work capacity, earning potential, and risk of chronic diseases in adulthood[2-4]. Childhood stunting is common among populations residing in adverse environments with limited access to nutrient-dense foods and poor sanitation[5]. In these environments, children's diets are predominantly cereal-based, with low amounts of animal source foods (ASFs) that contain high quality complete protein, and do not meet the high nutrient needs of young children[6]. Improving the quality of early childhood diets and reducing child stunting is therefore a public health priority in India.

Previous evidence based on randomized controlled trials (RCTs) evaluating supplementation with cereal legume-based complementary foods or single or multiple micronutrient interventions in children have shown a limited impact on linear growth[7-9]. The emerging evidence suggests that locally available ASFs like eggs could play an important role in improving linear growth of young children[10]. However, the evidence is mixed, and the impact of an egg intervention on linear growth and stunting prevalence appears to vary in different settings depending on the dietary patterns and baseline prevalence of child stunting. For example, an RCT in 163 children (6–9 months) from Ecuador assessed the impact of egg supplementation (1 egg per day for 6 months) and showed a reduction in the prevalence of stunting by 47% and underweight by 74% in the intervention arm (n=83)[11]. However, in a similar trial in 660 children (6-9 months) from Malawi, an egg intervention did not have a beneficial effect on linear growth, possibly due to low baseline prevalence of stunting and background diets being rich in ASFs[12]. In another RCT from Malawi (9 month old children, N=267), the addition of egg powder with bovine colostrum to complementary feeds resulted in lower prevalence of stunting in the intervention arm (n=135, RR = 0.70; 95% CI: 0.52, 0.94)[13]. Interventions such as preventive small-quantity lipid-based nutrient supplements for children aged 6-23 months, have shown positive impact on child growth[14]. Compared to such interventions, a locally available animal source food such as egg[15] will be cost effective and scalable. The aetiology of stunting is complex and poorly

Confidential: For Review Only

understood and the effect of interventions may differ between countries and ethnicities. There is no evidence available from the Indian subcontinent, where the stunting prevalence is high, and the ASF intake is low.

Eggs provide several nutrients critical to infant growth and development which include all the essential amino acids making it a high-quality/complete protein, docosahexaenoic acid (DHA), and micronutrients such as vitamin B12 which are important for neural development and immune function[10]. Eggs are among the richest sources of choline, which is necessary for the production of phospholipids, cell membrane integrity, and the conversion of acetylcholine and sphingomyelin necessary for brain development and function[16]. Dietary choline intake can modulate DNA methylation, therefore, egg supplementation may also impact the epigenetic signatures[17]. Epigenetics is one of the candidate mechanisms linking early life factors to physical and cognitive development with consequences across the life course[18-21]. Evaluation of epigenetics signatures linked with stunting is central to the Action Against Stunting Hub (AASH) observational cohort[22].

In India, eggs are more affordable than other ASFs and are relatively simple to store and prepare. Egg supplementation is a promising option in government nutrition programs. In recent years, a few states in India have included eggs in supplementary feeding programs for pregnant women and children in the age group 7 months to 6 years[23 24]. However, systematic impact evaluation of these interventions on anthropometric, biochemical, or cognitive outcomes has not been done. We hypothesize that daily egg supplementation (accompanied by behaviour change communication (BCC) to increase compliance to the intervention) starting at 9 months will improve linear growth and reduce the incidence of stunting at 18 months of age.

The proposed ENRICH study aims to evaluate the effect of daily supplementation with an egg in children from 9 months to 18 months of age (accompanied by behaviour change advice to the mother to increase compliance with the intervention) on:

1) linear growth and stunting at 18 months
2) cognition, nutritional status, and relevant epigenetic signatures.

Confidential: For Review Only

Methods and analysis

Study design and setting

The ENRICH study is a two-arm individually randomized open-labelled controlled trial nested within the AASH cohort study [25] (Figure 1). The study will be conducted in catchment areas of three primary health centres (Addagutta, Warasiguda and Sitaphalmandi) in Hyderabad, India which are within a vicinity of 5 km and have similar demographic and socioeconomic composition. These slums have a total population of ≈175,000 and previous studies in this area have found a high prevalence of childhood stunting (around 27% at 24months age)[9]. It was also reported that the caloric intakes of 18 month old children in this area were comparable to the National data of 1-3 year old children with rural background. Women will be recruited in the study in the third trimester of pregnancy, and the egg intervention will start when the children are 9 months old. Data and sample collection will be conducted as per the observational study protocol, including infant blood samples at 6 and 18 months; anthropometric measurements, dietary data and infant cognitive function at 9, 12 and 18 months of age. Blinding the participants is not possible due to the nature of the intervention, but the study staff measuring study outcomes will be blinded to the intervention group[25].

Sample size

Assuming α = 0.05 and 90% power and a difference of 0.32 in the length-for-age z score (LAZ) between the intervention and control groups with standard deviation (SD)= 0.70 and an allocation ratio of 1:4 in the intervention and control arms, accounting for a 10% loss to follow up, the estimated sample size is 69 children (rounded off to 70) in the intervention group and 277 (rounded off to 280) children in the control arm. This sample size is also powered (80%) to detect a difference of 0.5 SD in the cognitive outcomes.

The estimated effect size is higher than the other trials on complementary feeding interventions and is a conservative estimate of half the effect size in a similar study carried out in Ecuador[11]. The higher effect sizes are assumed due to the intensively supervised

Confidential: For Review Only

nature of the intervention delivery, longer duration of intervention and compliance-targeted behaviour change communication which is likely to result in higher compliance. For comparison of blood biomarkers relevant for the egg intervention, a random sub-sample of 70 children in the control group will be selected. This sample size is adequate to detect a difference of 0.35 µg/mL in choline as observed in the above study in Ecuador with about 90% power.

Outcome measures

The primary study outcome is the LAZ score at 18 months. The secondary outcomes include cognition measured by INTER-NDA component of the INTERGROWTH. (The INTER-NDA scale has been selected since these assessments are rapid (15 minutes), low-cost, and constructed specifically for use in low and-middle-income (LMIC) setting and can be administered by non-specialists. It has strong agreement with the Bayley's Scale for Infant Development-III (interclass correlation coefficients 0·75 to 0·88, p<0·001 for all domains). The tests are designed to be free from cultural biases and the kit consists of common household items. The other secondary outcome measurements include plasma concentrations of insulin-like growth factor 1(IGF-1), insulin-like growth factor binding protein-3 (IGFBP3), serum/plasma concentrations of essential amino acids, micronutrient status including; retinol, folate, vitamin B12, betaine, as well as red blood cell (RBC) fatty acids, DHA, choline and epigenetic (DNA methylation) signatures linked to the study outcomes. The detailed methodology has been described elsewhere[25].

Participant recruitment

The present trial is nested within the AASH observational cohort study where pregnant women (n=700) in the third trimester of pregnancy residing in the urban slums are being enrolled through the primary health centres (PHC) after eligibility assessment and obtaining written informed consent. The data collection in pregnancy and during the follow up of the offspring has been described elsewhere[25]. Of these, 350 children born from February 2022 to March 2023 will be a part of the present trial; 70 children will be randomized to the

Confidential: For Review Only

intervention arm and 280 to the control arm. The control arm children will also be a part of the observational cohort.

Eligibility

The inclusion criteria during pregnancy will be: healthy pregnant women in the age group 18-40 y, gestation 26-32 weeks, singleton pregnancy and willingness to comply with the study requirements. Children who were part of the AASH observational cohort and who attained 9 months at the inception of the ENRICH trial in November 2022 will be included. Out of these children, those who will relocate to a new area within next 9 months, those with a history of egg allergies (including family history), children with congenital anomalies or chronic morbidities, children whose parents were not willing to feed egg to their babies will be excluded from the study.

Randomization, intervention allocation and blinding

Each month, all children between 7.5 and 8.5 months of age will be randomly assigned to either the intervention or control group in a ratio of 1:4 after ascertaining the eligibility and obtaining written informed consent from the mother. Project staff collecting data on study outcomes will be blinded to the allocation sequence.

Through a centralised block randomization system, 350 children will be randomly allocated to blocks of 5 or 10 children, maintaining a ratio of 1:4 intervention and control children within each block. Allocation is concealed by sequentially numbered, opaque sealed envelopes.

Trial intervention, intervention delivery, and quality control

Egg supplementation will start on completion of 9 months of child age. The participant duration in the trial is for 9 months (till 18 months of age of the child). One hen's egg (medium-sized, weighing around 50 g, raw / boiled depending on the mother's choice) will be delivered to children’s homes every day by project staff, and mothers will be asked to feed it to the child within four hours. The mothers will be provided instructions on age-appropriate egg-based complementary food recipes through a purpose-made video during the first visit. The importance of feeding the egg to the index child and not sharing it with

Confidential: For Review Only

other family members will also be stressed during monthly counselling sessions to improve adherence to the intervention. This is a model of BCC developed based on evidence generated through best practices for Take –home rations of supplementary nutrition by Government of India[26]. The specific messages were developed through formative research conducted with parents and grandmothers of young children. The project staff will observe the first feeding episode of complementary food containing eggs, and mothers will be counselled in case of minor gastric problems such as cramps in the stomach, nausea and vomiting.

Study staff will visit the households twice a week to observe the child feeding in the first month of the intervention and will encourage and support the mothers. Information on consumption of an egg by the child on the previous day is collected daily by the field staff while delivering the eggs in the intervention group. For comparison, data on eggs consumption will also be collected every month during a home visit in the control group children.

The eggs will be procured locally from a verified supplier. The quality control measures to ensure food safety of the intervention will include periodic assessment of the microbial contamination of eggs: both at the point of purchase and the point of use. Every month, random sub-samples of eggs collected soon after purchase (3 raw and 3 boiled eggs) and complementary food prepared using eggs for the child (from 6 households every month) will be analysed for microbial contamination (Salmonella, Shigella, total coliform count) at the NIN laboratory. Eggs will be randomly selected for assessing nutrient composition for major nutrients (energy, proteins, iron, folate, calcium, vitamin A, D etc) every three months at the food chemistry laboratory of ICMR- NIN.

Control group

The state government currently provides eggs (16 eggs per month for 7 months to 3 years) for beneficiaries of Integrated child development services (ICDS, a Government initiative for all women and children who can voluntarily enrol) as a part of the Rations. However, during formative research with mothers(n=100), it was found that children below one year of age are not fed whole eggs due to fear of indigestion and other reasons. Moreover, 59% of children were not fed eggs supplied through the government's supplementary nutrition programs due to intra-household sharing[27] . Though there will not be any active

https://mc.manuscriptcentral.com/bmjpo

Confidential: For Review Only

intervention by the study team in the control group, all children from both the intervention and control groups may receive eggs as a part of Take-Home Ration, which may reduce the magnitude of the observed effect of the intervention. Also, there is a possibility of e 'contamination' of BCC messages in the control group in this individually randomized trial.

The number of eggs received through Government programme and the number of eggs consumed by the child will be recorded for the intervention as well as control group. We will assess the mother's knowledge of the nutritional benefits of eggs in both the groups at baseline and 18 months for comparison between the two groups.

Data collection at baseline and during follow up

Data collection in both the intervention and control groups would be done following the same protocol as the observational cohort. The parameters to be measured at different time points are listed in Table 1. Details on the methodology for assessing primary and secondary outcomes are described elsewhere[25]. A trained phlebotomist will draw a 3 ml venous blood sample at both six months and 18 months in children from the intervention group and a sub-sample of 70 children from the control groups. The methods for assessment of blood parameters and epigenetic signatures linked with stunting and cognition have been described elsewhere[22 25]. Data on morbidity (including information on episodes of hospital admission and sick-child clinic visits) will be collected using a structured questionnaire every two weeks. Mothers will be encouraged to report any adverse events to the study staff. Data will be collected using the android interface of commcare version 2.53 using Samsung A8 tab. The collected data will be securely stored in the database using encrypted codes. For data quality, we have used closed end questions wherever possible, and mandatory fields for core questions. Regex functionality is enabled for anthropometry data. The double data entries are manually managed at the supervisor level.

Withdrawal from the study

Confidential: For Review Only

During the consent process, mothers will be informed that they may choose to withdraw their child from the study at any time. Children whose mothers wish to withdraw after the randomization will be visited by a project staff to discuss and record the reasons for withdrawal.

In case of suggestive of egg allergy symptoms, the participants would be referred to the PHC, and the mother would be advised to consult the medical officer and seek advice on the continuation of egg supplementation. Data on the prevalence of egg allergy in Indian children is not available but based on the self-reported prevalence of egg allergy (< 2.5%) in other settings[28], around 1-2 cases are anticipated.

If a participant withdraws from the trial, data captured will contribute to the analysis up to the day prior to discontinuation, unless the mother wishes to withdraw consent for any data captured up to that point. Participants who withdraw from the study will not be replaced.

Statistical analysis

We will summarise demographic, clinical, and laboratory data using descriptive statistics. For missing data, multiple imputation method will be used[29]. The primary analysis will be based on the intention to treat (ITT) population and will compare the children's LAZ scores at the age of 18 months in the intervention and control groups using multilevel regression models. We will adjust the models for key covariates (E.g., age, gender and area) associated with the outcome variables. We will check the model assumptions and make appropriate adjustments to the analysis where necessary. We will also use difference-in-difference analysis to evaluate the impact of the intervention. We will compare the secondary outcome measures between the two groups using ITT principles. The study is powered for detecting meaningful differences in linear growth and DHA concentrations in the two groups. Other secondary outcome analyses will be exploratory. Additional analysis of the primary and secondary endpoints will also be presented using the per protocol population. No interim analysis is planned[30 31].

Confidential: For Review Only

Patient and Public Involvement

Patients were not directly involved in the design or conduct of the study. Formative research involving mothers, grandmothers and others from the local community (excluding study participants) assisted in understanding local cultural practices and developing BCC material [27].

Ethics and dissemination

This protocol, informed consent documents, and patient information sheets have been reviewed and approved by the Research Ethics Committees at ICMR-NIN (CR/3/IV/2022 dated 28.7.2022) and LSHTM (IRB no. 18029) as well as by an independent Data Safety Monitoring Board (DSMB). The trial is registered with the Clinical Trials Registry of India (CTRI/2021/11/038208). The DSMB is independent from the sponsor and competing interests and will review clinical data at regular intervals as the study progresses.
The study results will be disseminated to the local and national government departments (for potential modifications in the supplementary nutrition program); academic channels like national conferences and published in peer-reviewed scientific journals.

Significance and impact

The objectives of the present trial are well aligned with the objectives of the National Nutrition Mission in India, with the potential for immediate scale-up of the intervention. The study has several strengths. Chief among them is the comprehensive evaluation of outcome indicators ranging from linear growth, cognition, nutritional biomarkers and epigenetic states indicative of exposures and biological function. Additional information on maternal diets and blood biomarkers in pregnancy as well as information on the intrauterine growth of the offspring would allow us to examine pathways for the impact that previous studies have not

Confidential: For Review Only

reported. A limitation is that in this community-based intervention, egg consumption by the index child cannot be supervised completely. Moreover, '''contamination' of the BCC messages in the control group and contamination related to the Government's supplementary nutrition programme may attenuate the effect size. Nevertheless, if the trial demonstrates significant benefits, the evidence on the impact of this relatively inexpensive yet nutrient rich ASF in early life will inform strategies for stunting reduction in India and other developing countries.

Funding

The study is funded by the UK Research and Innovation, Global Challenges Research Fund (Grant No. EPPHZP5519).
.

'Authors' contributions:

SKB, SRC, BK: Conceptualized, designed the study and wrote first draft

LA, TD, DP, MC, KS, PA: developed detailed protocols, planned data collection, critical input to manuscript

RM, HD, CH, SFR, RNP, RP, RRK, NKR, MJ, PH: concept, design, critical inputs to manuscript

References

1. Comprehensive National Nutrition Survey (CNNS) National Report. Population Council: Ministry of health and family welfare, Government of India, UNICEF, 2018.
2. Hoffman DJ, Sawaya AL, Verreschi I, et al. Why are nutritionally stunted children at increased risk of obesity? Studies of metabolic rate and fat oxidation in shantytown children from Sao Paulo, Brazil. *The American journal of clinical nutrition* 2000;72(3):702-07.
3. Martins VJ, Toledo Florêncio TM, Grillo LP, et al. Long-lasting effects of undernutrition. *International journal of environmental research and public health* 2011;8(6):1817-46.

Confidential: For Review Only

4. Leroy JL, Frongillo EA. Perspective: What Does Stunting Really Mean? A Critical Review of the Evidence. *Advances in nutrition (Bethesda, Md)* 2019;10(2):196-204. doi: 10.1093/advances/nmy101 [published Online First: 2019/02/26]
5. World Health Organization. Reducing stunting in children: equity considerations for achieving the global targets 2025. Geneva: World Health Organization, 2018.
6. Kulkarni B. Addressing the double burden of malnutrition in developing countries: Need for strategies to improve the lean body mass. *Food and nutrition bulletin* 2018;39(2_suppl):S69-S76.
7. Christian P, Shaikh S, Shamim AA, et al. Effect of fortified complementary food supplementation on child growth in rural Bangladesh: a cluster-randomized trial. *International journal of epidemiology* 2015;44(6):1862-76.
8. Lassi ZS, Das JK, Zahid G, et al. Impact of education and provision of complementary feeding on growth and morbidity in children less than 2 years of age in developing countries: a systematic review. *BMC public health* 2013;13(3):1-10.
9. Radhakrishna K, Hemalatha R, Geddam JB, et al. Effectiveness of zinc supplementation to full term normal infants: a community based double blind, randomized, controlled, clinical trial. *PloS one* 2013;8(5):e61486.
10. Lutter CK, Iannotti LL, Stewart CP. The potential of a simple egg to improve maternal and child nutrition. *Maternal & child nutrition* 2018;14:e12678.
11. Iannotti LL, Lutter CK, Stewart CP, et al. Eggs in early complementary feeding and child growth: a randomized controlled trial. *Pediatrics* 2017;140(1)
12. Stewart CP, Caswell B, Iannotti L, et al. The effect of eggs on early child growth in rural Malawi: the Mazira Project randomized controlled trial. *The American journal of clinical nutrition* 2019;110(4):1026-33.
13. Bierut T, Duckworth L, Grabowsky M, et al. The effect of bovine colostrum/egg supplementation compared with corn/soy flour in young Malawian children: A randomized, controlled clinical trial. *The American Journal of Clinical Nutrition* 2021;113(2):420-27.
14. Keats EC, Das JK, Salam RA, et al. Effective interventions to address maternal and child malnutrition: an update of the evidence. *The Lancet Child & adolescent health* 2021;5(5):367-84. doi: 10.1016/s2352-4642(20)30274-1 [published Online First: 2021/03/11]
15. Papanikolaou Y, Fulgoni VL, 3rd. Eggs Are Cost-Efficient in Delivering Several Shortfall Nutrients in the American Diet: A Cost-Analysis in Children and Adults. *Nutrients* 2020;12(8) doi: 10.3390/nu12082406 [published Online First: 2020/08/17]
16. Iannotti L, Lutter C, Bunn D, et al. Eggs: The uncracked potential for improving maternal and young child nutrition among the world's. *Issue: Nutrition Reviews, 72 (6)* 2014
17. Zeisel SH. Choline, other methyl-donors and epigenetics. *Nutrients* 2017;9(5):445.
18. Tiffon C. The impact of nutrition and environmental epigenetics on human health and disease. *International journal of molecular sciences* 2018;19(11):3425.
19. Haggarty P, Ferguson-Smith AC, Kuh D, et al. Life course epigenetics and healthy ageing. *A life course approach to healthy ageing* 2013:198-212.
20. Lorgen-Ritchie M, Murray AD, Staff R, et al. Imprinting methylation predicts hippocampal volumes and hyperintensities and the change with age in later life. *Scientific reports* 2021;11(1):1-12.
21. Lorgen-Ritchie M, Murray AD, Ferguson-Smith AC, et al. Imprinting methylation in SNRPN and MEST1 in adult blood predicts cognitive ability. *Plos one* 2019;14(2):e0211799.

Confidential: For Review Only

22. Anouschka R, Magatte N, Rajender RaoKalashikam et al. Epigenetic Studies in Families and Children at Risk of Stunting in India, Indonesia, and Senegal. BMJ pediatrics Open, 2022.
23. Kulkarni B, Mamidi R, Balakrishna N, et al. Body composition assessment in infancy and early childhood: comparison of anthropometry with dual-energy X-ray absorptiometry in low-income group children from India. *European journal of clinical nutrition* 2014;68(6):658-63.
24. Department of women and child development. Government of Telangana, 2022.
25. Davies-Kershaw H, Fahmida U, Kyaw HM, et al. Anthropometric, biochemical, dietary, morbidity and wellbeing assessments in women and children in Indonesia, India and Senegal: a UKRI GCRF Action Against Stunting Hub protocol paper. BMJ Pediatrics Open, 2022.
26. NITI Aayog and World food programme. Take home ration: Good practices across the States/UTs: NITI Aayog, 2022.
27. Thomas N, Kulkarni B, Augustine L, et al. Formative Study to Identify the Determinants, Current Practices and Knowledge of Mothers related to the Egg Consumption of Young Children in Hyderabad, Telangana. Abstract No. OP-2022-0098. 54th Annual conference of Nutrition Society of India. Hyderabad: ICMR-National Institute of Nutrition, Hyderabad, 2022.
28. Muraro A, Halken S, Arshad S, et al. EAACI food allergy and anaphylaxis guidelines. Primary prevention of food allergy. *Allergy* 2014;69(5):590-601.
29. Kang H. The prevention and handling of the missing data. *Korean journal of anesthesiology* 2013;64(5):402-6. doi: 10.4097/kjae.2013.64.5.402 [published Online First: 2013/06/07]
30. McCoy CE. Understanding the Intention-to-treat Principle in Randomized Controlled Trials. *The western journal of emergency medicine* 2017;18(6):1075-78. doi: 10.5811/westjem.2017.8.35985 [published Online First: 2017/11/01]
31. Rosenblum M. Robust Analysis of RCTs Using Generalized Linear Models. Targeted Learning: Springer 2011:187-99.

Confidential: For Review Only

Table 1: Variables measured at different time points

Confidential: For Review Only

Measure	**Timelines** (intervention and control group)
**Biological sample/ Biomarkers**	
Buccal sample for epigenetics	At birth and at 18 months
**Plasma:** Micronutrient status (Hemoglobin, serum ferritin, serum transferrin receptors, growth hormone, serum iron, zinc, retinol, folate, vitamin B12, folate, IGF1, IGFBP3)	At 3rd trimester of pregnancy and at 6 and 18 months
Blood biomarkers relevant for egg intervention: RBC fatty acids, amino acids, choline, betaine	At 6 and 18 months (in the intervention group and in a sub-sample of 70 children from control group)
Inflammatory markers in blood: CRP and AGP	At 6 and 18 months
**Stool sample:** for entero-pathogens, parasites, and inflammatory markers MPO, AAT	At 6 and 18 months
Micronutrient content of the breastmilk samples: vitamin A, B12, folate	At 3 months postpartum
**Diet, anthropometry and cognition**	
**Diet**<br>• Multiple pass24-hour diet recall with repeat in 10% of the sample<br>• Initiation of breast feeding<br>• Exclusive breastfeeding and introduction to complementary foods	Dietary 24-hour recall at 6, 9, 12 and 18 months<br>Breast feeding questionnaire at birth<br>Breastfeeding questionnaire at 1-6 months

Confidential: For Review Only

**Anthropometry measurements**<br>Standing and sitting height, weight, skinfolds and MUAC<br>Length, weight, Head circumference, MUAC, knee-heel length and skinfolds	At 3rd trimester of pregnancy and at 3,6,9,12, 18 and 24 months at birth. Only weight only at 1, 2, 4 and 5 months.
**Assessment of cognition with context specific tests**<br>Home Observation Measurement of the Environment (HOME -Scale)<br>Oxford Neurodevelopment Assessment (OX-NDA)<br>NTER- Neurodevelopment assessment (9INTER-NDA):	At 10-14 months and 18 months<br>At 10-14 months<br>At 22-24 months
**Assessment of morbidity** using structured pictorial charts to be completed daily	Completed by mother each week from birth until 18 months every two weeks throughout the follow up
**Assessment of home environment**	Using a structured questionnaire at 9 months

IGF1: Insulin like growth factor-1; IGFBP3: Insulin like growth factor binding protein 3; CRP: c-reactive protein; AGP: alpha 1-gycoprotein

MUAC: Mid upper arm circumference; MPO: Myeloperoxidase, AAT: alpha-1-antitrypsin

Confidential: For Review Only

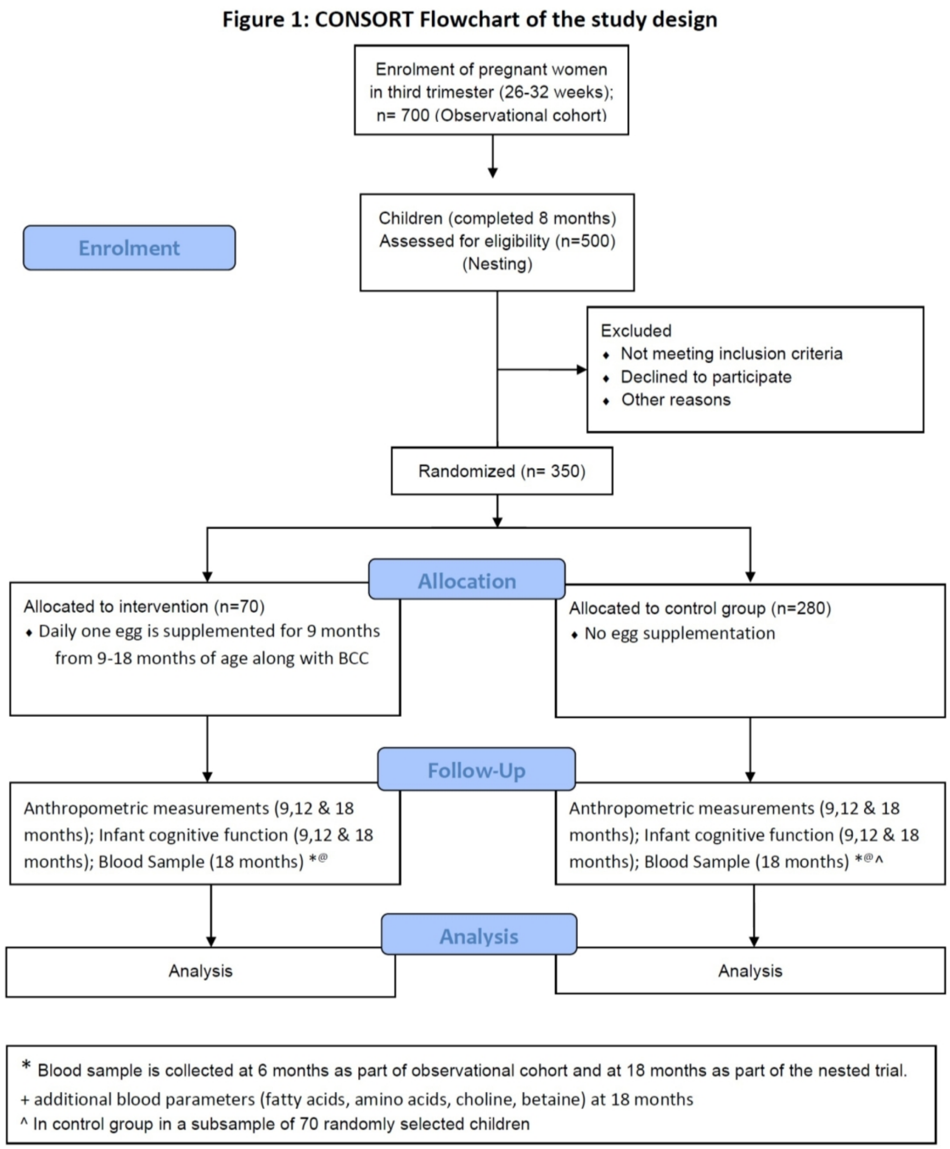

Figure 1: CONSORT Flowchart of the study design

176x214mm (300 x 300 DPI)

Appendix 1: Questionnaires for data collection

1. Egg preparation and delivery

2) Date and time of the cooking (Date and time)

3) When it was delivered (Time)

4) Did the child receive the egg with in the time (4 h from cooking time)?

   a) Yes

   b) No

4) If no, what is the reason

   1. Traffic

   2. The mother/ family members were not available at home

   3. The mother/ family members rejected the egg

   96. Other specify

2. Daily Compliance questionnaire

1 Did the child receive the egg yesterday

   1. Yes

   2. No

2 If yes, did the child consume the egg yesterday

   1. Yes

   2. No

3 Which portion of the egg is given to the child?

   1. Only egg white

   2. Only egg yolk/yellow

   3. Whole egg

4 At what time the child consumed the egg

   Date and time (recorded)

6 If No, why was it not consumed yesterday

   1. The child disliked the taste

   2. The child fell sick/illness/diarrhoea/vomiting

   3. Family members/others advised not to give

   96. Other, specify

Confidential: For Review Only

3. Monthly follow-up questionnaire (Control)

1. Are you getting eggs from AWC: yes/No
2. If yes how many eggs did you receive last month?
   a. 16 b. >16 c.<16
3. Have you started egg feeding: Yes/No
4. If Yes, how frequently eggs were consumed in the previous week
   a. Everyday b. 4-6 days c. 1-3 days d. Never
5. What is the portion size usually eaten by the child
   a. >1 egg. B. 1 egg. C. ½ egg. D. ¼ egg
6. Was the egg mixed with other food for consumption: Yes/ No
7. Number of times egg eaten per day
   a. Once. B. 2 times. C. 3 times. D. More than 4 times
8. Which portion of the egg was readily accepted by the child:
   a. Yolk. B. White. C. Whole D. None
9. Did your child experience any of the following difficulties after egg consumption
   a. Fever. B. Cough. C. Vomitings& Diarrhoea. D. Wheezing. E. tooth ache F None
10. Have you noticed any change in the child's appetite
    a. Yes, increased. B. Yes, Decreased. C. No change
11. In the previous week have you fed eggs procured from any other sources:yes/No
12. If yes, mention the source___________

Confidential: For Review Only

4. Pre-intervention questionnaire (knowledge and practice), (in intervention and control at endline; only in intervention at the beginning and at midline)

PART 1: PRACTICE

1. Do you currently breastfeed your child?
   a. Yes
   b. No

2. How many times do you breastfeed the child in a day? (Duration of breastfeeding should be at least 10 minutes)
   a. Less than 4 times
   b. 4 times
   c. 5-6 times
   d. More than 6 times

3. Do you give complementary food to your child? [Dropdown]
   a. Yes
   b. No

4. At what age did you initiate complementary feeding to your child?
   a. Less than 6 months
   b. 6 months
   c. 7 months
   d. 8 months
   e. Not yet started,
   f. Other, specify_________

5. If not yet started, what is the reason for not starting complementary feeding to your child?
   a. Child is too small to eat solid foods
   b. Child doesn't have teeth to chew food
   c. Elders advised not to
   d. Fear of indigestion

6. What are the types of complementary foods that are currently given to the child? [Multiple options can be selected]
   a. Not yet started
   b. Formula milk (Eg: Lactogen,amul milk spray etc . . .)
   c. Commercial baby foods (Eg: Ceralac, farex,nestem etc. . .)
   d. Homemade semi-solids (Eg: Uggu, rice+ dhal (soft cooked), mash fruits&vegetables etc. . .)
   e. Processed food (Eg:Biscuits,kurkure,chips etc . . .)

Confidential: For Review Only

f. Homemade solids (Eg: Normal food)
g. Other, specify_______

7. What are the foods that are generally included in the homemade complementary foods? (logic)[Multiple options can be selected]
a. Cereals & millets
b. Pulses
c. Green leafy vegetables
d. Roots & tubers
e. Other vegetables
f. Fruits
g. Flesh foods
h. Eggs
i. Milk & milk products
j. Fat & oils
k. Sugar & jaggery.

8. Do you feed eggs to your child? Skip Logic (Questions regarding feeding egg, only if the mother is feeding egg to her child)
a. Yes
b. No

9. If yes, at what age did you start feeding eggs to your child?
a. 6 months
b. 7 months
c. 8 months
d. 9 months
e. Other, specify _________

10. If not, what is the reason for not initiating egg to the child before 9 months?
a. The eggs do not get digested
b. The eggs cause heat build-up in the child's body
c. Family members suggested to avoid eggs till 8th month
d. Eggs are high in fat/cholesterol
e. Other, specify_________________

11. Who suggested not to initiate egg to your child?
a. Husband
b. Mother-in-law
c. Neighbours
d. Doctor
e. Other, specify ____________

12. What form of the egg did your child like to eat?
a. Boiled Egg
b. Half boiled
c. Omelette /Scrambled Egg
d. Egg Curry
e. Mashed with rice
f. Other, specify ____________

Confidential: For Review Only

13. Did you stop feeding eggs to your child after initiating?
    a. Yes
    b. No

14. If yes, why did you stop feeding eggs to your child?
    a. The child disliked the taste
    b. The child had an allergy
    c. The child had diarrhoea/vomiting
    d. Family members/others advised not to give.
    e. Other, specify___________________

15. If the child did not like the taste, what did you do?
    a. Tried a different recipe with egg
    b. Stopped feeding egg
    c. Other, specify_____________

16. If the child developed an allergy/diarrhoea/ vomiting after consuming the egg, whom did you contact?
    a. Government hospital / PHC doctor
    b. Private doctor/Registered Medical Practitioner
    c. Local medical shop
    d. Traditional/ Religious leaders
    e. Treated at home
    f. Family members/neighbours suggested
    g. Other, specify _____________

17. Does any member of the family have an allergy to any food items?
    a. Yes
    b. No

18. Who has an allergy to the food item?
    a. Father of the child
    b. Mother of the child
    c. Grandparents of the child
    d. Siblings

19. Which food is allergic to the above-mentioned person?
    ____

20. How do you feed eggs to your child? [MCQ- Multiple options can be selected]
    a. Mashed with rice
    b. Boiled Egg
    c. Half boiled
    d. Omelette /Scrambled Egg
    e. Egg Curry
    f. Other, specify __________________

21. Which part of the egg is given to the child? [Drop down]

Confidential: For Review Only

a. Only egg white
b. Only egg yolk
c. Whole egg

22. Why do you give only the white part of the egg to the child? [If selected a]-
a. Child likes the taste
b. Easy to swallow
c. Family members / elders advised
d. Doctor advised
e. ASHA/ ANM/Anganwadi worker advised.
f. Other, specify_________________

23. Why do you give only the yellow part to the child? [If selected b]
a. Child likes the taste
b. Easy to swallow
c. Family members/elders advised
d. Doctor advised
e. ASHA/ ANM/Anganwadi worker advised.
f. Other, specify _________________

24. How frequently do you feed eggs to your child? [Drop down]
a. Daily
b. Twice a week
c. Thrice a week
d. >4 times a week
e. Weekly once

25. Usually, how many eggs do you feed your child in a day? [MCQ]
a. One whole egg
b. Half egg
c. Two whole eggs
d. One whole egg white
e. Two whole egg white
f. One whole egg yolk
g. Two whole egg yolk
h. Other, specify___________________

26. If the egg is not fed -, please tell us the reason from the options provided below [Multiple options can be selected]
a. Not available and accessible
b. The child dislikes eating egg
c. No time to cook the egg
d. Family members do not prefer
e. Other, specify__________________

27. Do you avoid feeding eggs to your child on festivals/special days in a week? [Skip Logic-If yes, go to Q. 21, If not, go to 23]
a. Yes
b. No

28. If yes, please mention on which days in the week do you avoid feeding eggs to the child? [Short answer]

Confidential: For Review Only

____

29. If yes, please mention on which occasions do you avoid feeding eggs to the child? (Prompt: Festivals, seasons) [Short answer]

____

Confidential: For Review Only

PART 2: KNOWLEDGE

This section is to know the knowledge of the mother regarding feeding egg to the child

30. Are eggs an important component of the child's diet for their growth and development [Drop down]
    a. Yes
    b. No
    c. Don't know

31. What are the benefits of feeding an egg to the child? [Multiple options can be selected]
    a. Eggs are good for vision
    b. Eggs help the child grow tall
    c. Eggs provide immunity
    d. Eggs are good for bones and teeth
    e. Eggs increase brain capacity
    f. All of the above
    g. None of the above
    h. I don't know
    i. Other, specify________

32. Which part of the egg is healthy for the child? [Drop down]
    a. Only Egg yolk
    b. Only Egg white
    c. Whole egg
    d. Don't know

33. At what age the children should start consuming eggs? [Drop down]

    ____

34. I intend to feed one whole egg daily to the child from 9 months of age [Drop down]
    a. Extremely unlikely
    b. Unlikely
    c. Neutral
    d. Likely
    e. Extremely likely

35. I am confident that I can feed one whole egg to the child every day from 9 months of age [Drop down]
    a. Not confident
    b. Confident
    c. Very confident
    d. Neutral

36. Husband and elders in the family will support in feeding egg to the child from 9 monthsof age [Drop down]

Confidential: For Review Only

a. Agree
b. Disagree
c. Don't know

37. Would you like to know about the recipes you can prepare and feed your child? [Dropdown]
a. Yes
b. No

ICDS

38. Did you enrol your or your child's name in the local ICDS center/ Anganwadi? [SkipLogic- If not, go to 44]
a. Yes
b. No

38. a If yes, What are the benefits you are getting from the ICDS centre (Multiple choices can be selected)
a. Arogya Lakshmi one full meal (During pregnancy and lactation of 6 months)
b. Balamrutham
c. Eggs
d. Iron and folic tablets
e. Milk
f. Others, please specify___________________________

39. How many eggs do you receive from the ICDS center in a month?
a. 16
b. Less than 16
c. Don't know /remember

40. Have you received less than 16 eggs anytime from the ICDS center/ Anganwadi?
a. Yes
b. No

41. If yes, do you know the reason for receiving less than 16 eggs from the ICDS center?
a. No
b. Yes, specify

42. How frequently are the eggs given from the ICDS center are consumed by your child in the last 30 days? [Skip Logic-Skip next question, if selected Option. a, Go to next question if selected option. b./c./d.]
a. Often
b. Rarely
c. Never consumed

43. If the number of eggs fed is less than the allowance, what is the reason? [MCQ]
a. Not received or received less number of eggs from the center
b. The child is too young to eat the egg
c. Shared it with family members
d. Discarded
e. Other, specify ______

Confidential: For Review Only

44. Is there any organization/non-governmental organization (NGO) that sponsors eggs for your family?
    a. Yes
    b. No

45. If yes, what is the name of the organization? (Short answer)

____

46. What is the number of eggs that you receive from the organization mentioned above?

____

5. Preliminary screening (For both intervention and control)

1. Will you relocate to a new place in the next 9 months period?

a.Yes

b.No

c.Maybe

2.Do you give complementary food to your child?

a.Yes

b.No

3.Do you consume eggs at your home?

a. Yes

b.No

4.Have you started feeding eggs to your child?

a. Yes

b.No

5.Does your child have allergic reactions after consumption of egg?

a.Yes

b.No

c. Don't know

6.Have any other allergic reactions to any other food item occurred to your child?

Confidential: For Review Only

a. Yes

b. No

c. Don't know

7. Which food item is your child allergic to?

____

8. Is any of the family member allergic to any food items?

a. Yes

b. No

c. Maybe

9. Which family member has allergic reactions to food item?

a. Mother b. Father c. Siblings

d. Grandparents.

10. Which food item does the above-mentioned person has allergic reactions to?

____

11. Does your child have any of the below mentioned conditions?

a. Congenital anomalies/diseases

b. Chronic morbidity

c. None

12. Will you be willing to initiate egg consumption for your child and be a part of this egg supplementation group if randomly selected from 9 to 18 months of your child's age?

a. Yes

b. No

13. Did your child get MMR vaccination?

a. Yes

b. No

Confidential: For Review Only

Appendix 2

Protocol for Management of egg allergy

Since egg allergies typically present immediately or within two hours after ingestion, an auxiliary nurse midwife will be present during the first egg feed and she will observe the child for any for signs of egg allergies or anaphylaxis for a period of 2 hours. In case of allergy/anaphylaxis, allergies will be clinically evaluated by a paediatrician and treated clinically with a referral to tertiary hospital for management after giving a dose of antihistamine in the field unit of the study.

Second generation antihistamines and the dose will be kept with the Staff nurse for use in case of emergency.

-Cetirizine 6 months to < 2 years: 0.25 mg/kg;

- Levocetirizine 6 months to 5 years: 1.25 mg OD

Any child exhibiting egg allergy will be excluded from the study.

Confidential: For Review Only

BMJ Paediatrics Open

Eggs for Improving Nutrition, cognitive development and Reducing linear growth retardation among Infants and young Children (ENRICH): protocol of an egg supplementation trial among 9-18 months old children in Hyderabad, India

Journal:	*BMJ Paediatrics Open*
Manuscript ID	bmjpo-2023-001892.R2
Article Type:	Protocol
Date Submitted by the Author:	10-Jul-2023

Complete List of Authors:	Banjara, Santosh Kumar; National Institute of Nutrition, Clinical Division<br>Challa, Sai Ram; National Institute of Nutrition, Maternal and Child Health, Nutrition<br>Augustine, Little Flower; National Institute of Nutrition<br>Dasi, Teena; National Institute of Nutrition<br>Madhari, Radhika; ICMR - National Institute of Nutrition, Hyderabad, India<br>Fernandez Rao, Sylvia; National Institute of Nutrition<br>Palika, Ravindranadh; National Institute of Nutrition, Drug Safety Division; NATIONAL INSTITUTE OF NUTRITION<br>Pullakhandam, Raghu; National Institute of Nutrition<br>Kalashikam, Rajender Rao; National Institute of Nutrition<br>Kumar, Ramachandrappa Naveen; National Institute of Nutrition<br>Palepu, Dharani; National Institute of Nutrition, Clinical Division<br>Chilumula, Monica; National Institute of Nutrition, Maternal and Child Health, Nutrition<br>Selvaraj, Kiruthika; National Institute of Nutrition<br>Gopinath, Preethi; National Institute of Nutrition<br>Davies-Kershaw, Hilary; LSHTM, Department of Population Health; London School of Hygiene and Tropical Medicine<br>Jobarteh, Modou; London School of Hygiene & Tropical Medicine, Epidemiology & Population Health<br>Haggarty, Paul; University of Aberdeen<br>Heffernan, Claire; University of London, Department of Pathobiology and Population Sciences; London International Development Centre (LIDC)<br>Ferguson, Elaine; London School of Hygiene and Tropical Medicine (LSHTM)<br>Kulkarni, Bharati; National Institute of Nutrition

Keywords:	Nutrition, Epidemiology, Growth, Child Psychiatry, Health services research

I, the Submitting Author has the right to grant and does grant on behalf of all authors of the Work (as defined in the below author licence), an exclusive licence and/or a non-exclusive licence for contributions from authors who are: i) UK Crown employees; ii) where BMJ has agreed a CC-BY licence shall apply, and/or iii) in accordance with the terms applicable for US Federal Government officers or employees acting as part of their official duties; on a worldwide, perpetual, irrevocable, royalty-free basis to BMJ Publishing Group Ltd ("BMJ") its licensees and where the relevant Journal is co-owned by BMJ to the co-owners of the Journal, to publish the Work in this journal and any other BMJ products and to exploit all rights, as set out in our licence.

The Submitting Author accepts and understands that any supply made under these terms is made by BMJ to the Submitting Author unless you are acting as an employee on behalf of your employer or a postgraduate student of an affiliated institution which is paying any applicable article publishing charge ("APC") for Open Access articles. Where the Submitting Author wishes to make the Work available on an Open Access basis (and intends to pay the relevant APC), the terms of reuse of such Open Access shall be governed by a Creative Commons licence – details of these licences and which Creative Commons licence will apply to this Work are set out in our licence referred to above.

Other than as permitted in any relevant BMJ Author's Self Archiving Policies, I confirm this Work has not been accepted for publication elsewhere, is not being considered for publication elsewhere and does not duplicate material already published. I confirm all authors consent to publication of this Work and authorise the granting of this licence.

Confidential: For Review Only

Eggs for Improving Nutrition, cognitive development and reducing linear growth retardation among Infants and young Children (ENRICH): protocol of an egg supplementation trial among 9-18 months old children in Hyderabad, India

Santosh Kumar Banjara, Sai Ram Challa, Little Flower Augustine, Teena Dasi, Radhika Madhari, Sylvia Fernandez Rao, Ravindranadh Palika, Raghu Pullakhandam, Rajender Rao Kalashikam, Ramachandrappa Naveen Kumar, Dharani Prathyusha Palepu, Monica Chilumula, Kiruthika Selvaraj, Preethi Gopinath, Hilary Davies-Kershaw, Modou Lamin Jobarteh, Paul Haggarty, Claire Heffernan, Elaine Ferguson, Bharati Kulkarni

Corresponding author: Sai Ram Challa, MBBS, MD

Scientist 'E', Head, Clinical Division (Maternal& Child Health and Nutrition), ICMR-National Institute of Nutrition, Hyderabad, India

Email: drchallanin@gmail.com

Phone: +91-9618774603

Confidential: For Review Only

Abstract

Introduction

Evidence on the impact of nutrient-rich animal source foods like eggs for improving child growth and cognition is inconsistent. This study aims to examine the impact of an egg intervention in children, along with behaviour change communication (BCC) to the mother, on linear growth and cognition, and nutritional status in 9-18 month old children.

Methods and analysis

A 9-month open-labelled randomized controlled trial will be conducted in 3 urban slums in Hyderabad, India, as a sub-study of an observational cohort study (n=350) following pregnant women and their children until 18 months of age in a population at risk of stunting. The children born to women enrolled during the third trimester of pregnancy will be block randomized in a 1:4 ratio into the intervention (n=70) and control (n= 280) groups. Children in the intervention group will be supplemented with one egg per day starting from 9 months until 18 months of age. BCC designed to enhance adherence to the intervention will be used. The control group will be a part of the observational cohort and will not receive any intervention from the study team. The primary outcome will be length-for-age z-scores, and the secondary outcomes will include cognition, blood biomarkers of nutritional status including fatty acid profile, and epigenetic signatures linked with linear growth and cognition. Multivariate intention-to-treat analyses will be conducted to assess the effect of the intervention.

Ethics and dissemination

The study is approved by the Institutional ethics committees of ICMR-National Institute of Nutrition, Hyderabad, India and London School of Hygiene and Tropical Medicine, UK. The results will be published in peer-reviewed journals and disseminated to policymakers. Findings will also be shared with study participants and community leaders.

Trial Registration number: CTRI/2021/11/038208

Confidential: For Review Only

Key messages

What is known about the subject

- Previous studies using single or multiple micronutrients or cereal-legume based food supplements have shown only modest impact on child stunting.
- Evidence on the impact of nutrient dense animal source foods like eggs for improving child growth and cognition is inconsistent.

What this study hopes to add

- Comprehensive evidence on the impact of daily egg supplementation during 9 to 18 months of age on the linear growth, cognition, nutritional biomarkers and epigenetic outcomes

How this study might affect research, practice or policy

- Eggs provide several critical nutrients to support the infant growth and development and egg supplementation is a promising option for nutrition programmes.
- Therefore, the trial has a potential for immediate scale-up.

For Peer Review Only

Introduction

The high prevalence of stunting in children under five years of age in India (35%) remains a cause of concern given the associated long-term negative consequences on cognition, physical work capacity, earning potential, and risk of chronic diseases in adulthood. [1-4] Childhood stunting is common among populations residing in adverse environments with limited access to nutrient-dense foods and poor sanitation.[5] In these environments, children's diets are predominantly cereal-based, with low amounts of animal source foods (ASFs) that contain high quality complete protein, and do not meet the high nutrient needs of young children.[6] Improving the quality of early childhood diets and reducing child stunting is therefore a public health priority in India.

Previous evidence based on randomized controlled trials (RCTs) evaluating supplementation with cereal legume-based complementary foods or single or multiple micronutrient interventions in children have shown a limited impact on linear growth.[7-9] The emerging evidence suggests that locally available ASFs like eggs could play an important role in improving linear growth of young children.[10] However, the evidence is mixed, and the impact of an egg intervention on linear growth and stunting prevalence appears to vary in different settings depending on the dietary patterns and baseline prevalence of child stunting. For example, an RCT in 163 children (6–9 months) from Ecuador assessed the impact of egg supplementation (1 egg per day for 6 months) and showed a reduction in the prevalence of stunting by 47% and underweight by 74% in the intervention arm (n=83).[11] However, in a similar trial in 660 children (6-9 months) from Malawi, an egg intervention did not have a beneficial effect on linear growth, possibly due to low baseline prevalence of stunting and background diets being rich in ASFs.[12] In another RCT from Malawi (9 month old children, N=267), the addition of egg powder with bovine colostrum to complementary feeds resulted in lower prevalence of stunting in the intervention arm (n=135, RR = 0.70; 95% CI: 0.52, 0.94).[13] Interventions such as preventive small-quantity lipid-based nutrient supplements for children aged 6-23 months, have shown positive impact on child growth.[14] Compared to such interventions, a locally available animal source food such as egg will be cost effective and scalable.[15] The aetiology of stunting is complex and poorly understood and the effect of interventions may differ between countries and ethnicities.

Confidential: For Review Only

There is no evidence available from the Indian subcontinent, where the stunting prevalence is high, and the ASF intake is low.

Eggs provide several nutrients critical to infant growth and development which include all the essential amino acids making it a high-quality/complete protein, docosahexaenoic acid (DHA), and micronutrients such as vitamin B12 which are important for neural development and immune function.[10] Eggs are among the richest sources of choline, which is necessary for the production of phospholipids, cell membrane integrity, and the conversion of acetylcholine and sphingomyelin necessary for brain development and function.[16] Dietary choline intake can modulate DNA methylation, therefore, egg supplementation may also impact the epigenetic signatures.[17] Epigenetics is one of the candidate mechanisms linking early life factors to physical and cognitive development with consequences across the life course.[18-21] Evaluation of epigenetics signatures linked with stunting is central to the Action Against Stunting Hub (AASH) observational cohort.[22]

In India, eggs are more affordable than other ASFs and are relatively simple to store and prepare. Egg supplementation is a promising option in government nutrition programs. In recent years, a few states in India have included eggs in supplementary feeding programs for pregnant women and children in the age group 7 months to 6 years.[23 24] However, systematic impact evaluation of these interventions on anthropometric, biochemical, or cognitive outcomes has not been done. We hypothesize that daily egg supplementation (accompanied by behaviour change communication (BCC) to increase compliance to the intervention) starting at 9 months will improve linear growth and reduce the incidence of stunting at 18 months of age.

The proposed ENRICH study aims to evaluate the effect of daily supplementation with an egg in children from 9 months to 18 months of age (accompanied by behaviour change advice to the mother to increase compliance with the intervention) on:

1) linear growth and stunting at 18 months
2) cognition, nutritional status, and relevant epigenetic signatures.

Confidential: For Review Only

Methods and analysis

Study design and setting

The ENRICH study is a two-arm individually randomized open-labelled controlled trial nested within the AASH cohort study (Figure 1).[25] The study will be conducted in catchment areas of three primary health centres (Addagutta, Warasiguda and Sitaphalmandi) in Hyderabad, India which are within a vicinity of 5 km and have similar demographic and socioeconomic composition. These slums have a total population of ≈175,000 and previous studies in this area have found a high prevalence of childhood stunting (around 27% at 24months age).[9] It was also reported that the caloric intakes of 18 month old children in this area were comparable to the National data of 1-3 year old children with rural background. Women will be recruited in the study in the third trimester of pregnancy, and the egg intervention will start when the children are 9 months old. Data and sample collection will be conducted as per the observational study protocol, including infant blood samples at 6 and 18 months; anthropometric measurements, dietary data and infant cognitive function at 9, 12 and 18 months of age. Blinding the participants is not possible due to the nature of the intervention, but the study staff measuring study outcomes will be blinded to the intervention group.[25]

Sample size

The sample size (70 in intervention and 280 in control group) was calculated with 90 % power for an effect size of 0.32 in the length-for-age z score (LAZ) between the intervention and control groups with standard deviation (SD)= 0.70, drop-out of 10% and an allocation ratio of 1:4. This sample size is also adequate to detect 0.26 SD difference in LAZ between the two groups with 80% power and is also powered (80%) to detect a difference of 0.5 SD in the cognitive outcomes.

We believe that our study may result in a larger effect size (≥ 0.26 SD) than previous studies due to the intensively supervised nature of our intervention delivery which is likely to result in higher compliance to the supplement as compared to the previous studies. Our study intervention includes daily supply of ready to eat boiled egg in contrast to weekly delivery of raw eggs in Ecuador and Malawi studies,[11 12] longer duration of intervention (9

Confidential: For Review Only

months Vs 6 months' supply of eggs in previous studies), compliance targeted BCC including daily egg consumption record and an egg calendar. Recent studies on comprehensive supervised interventions have shown larger benefit (around 0.4 SD in LAZ) in improving linear growth faltering.[26] Moreover, an intensively supervised intervention needs to have a larger effect size to be considered for potential scale-up.

Outcome measures

The primary study outcome is the LAZ score at 18 months. The secondary outcomes include cognition measured by the Oxford Neurodevelopment Assessment (OX-NDA) at 12 months and the INTER-NDA (a multidimensional international assessment of child development) component of the INTERGROWTH- 21st Neurodevelopment Assessment. These tests have been selected since these assessments are rapid (15 minutes), low-cost, and constructed specifically for use in low and-middle-income (LMIC) setting and can be administered by non-specialists in field sites for a large sample.[27] It has strong agreement with the Bayley's Scale for Infant Development-III (interclass correlation coefficients 0·75 to 0·88, p<0·001 for all domains.[28] The OX-NDA showed moderate agreement for cognition and motor outcomes (ICCs 0.63 and 0.68, $p < 0.001$). The tests are designed to be free from cultural biases and the kit consists of common household items. The Home Observation for Measurement of the Environment (HOME) has been widely used to assess opportunities for cognitive stimulation at home setting.[29] The scale consists of 45 items categorized to six subscales: receptivity, acceptance, organisation, learning materials, engagement, and diversity. The researcher rates each item with a score of 1 if it occurred during the visit. Some items are also reported by the caregiver. Higher scores are indicative of better environment. The scale has been previously used in India.[30 31] The other secondary outcome measurements include plasma concentrations of insulin-like growth factor 1(IGF-1), insulin-like growth factor binding protein-3 (IGFBP3), serum/plasma concentrations of essential amino acids, micronutrient status including; retinol, folate, vitamin B12, betaine, as well as red blood cell (RBC) fatty acids, DHA, choline and epigenetic (DNA methylation)

https://mc.manuscriptcentral.com/bmjpo

Confidential: For Review Only

signatures linked to the study outcomes. The detailed methodology has been described elsewhere.[25]

Participant recruitment

The present trial is nested within the AASH observational cohort study where pregnant women (n=700) in the third trimester of pregnancy residing in the urban slums are being enrolled through the primary health centres (PHC) after eligibility assessment and obtaining written informed consent. The data collection in pregnancy and during the follow up of the offspring has been described elsewhere.[25] Of these, 350 children born from February 2022 to March 2023 will be a part of the present trial; 70 children will be randomized to the intervention arm and 280 to the control arm. The control arm children will also be a part of the observational cohort.

Eligibility

The inclusion criteria during pregnancy will be: healthy pregnant women in the age group 18-40 y, gestation 26-32 weeks, singleton pregnancy and willingness to comply with the study requirements. Children who were part of the AASH observational cohort and who attained 9 months at the inception of the ENRICH trial in November 2022 will be included. Out of these children, those who will relocate to a new area within next 9 months, those with a history of egg allergies (including family history), children with congenital anomalies or chronic morbidities, children whose parents were not willing to feed egg to their babies will be excluded from the study.

Randomization, intervention allocation and blinding

Each month, all children between 7.5 and 8.5 months of age will be randomly assigned to either the intervention or control group in a ratio of 1:4 after ascertaining the eligibility and obtaining written informed consent from the mother. Project staff collecting data on study outcomes will be blinded to the allocation sequence.[11]

Confidential: For Review Only

Through a centralised block randomization system, 350 children will be randomly allocated to blocks of 5 or 10 children, maintaining a ratio of 1:4 intervention and control children within each block. Allocation is concealed by sequentially numbered, opaque sealed envelopes.

Trial intervention, intervention delivery, and quality control

Egg supplementation will start on completion of 9 months of child age. The participant duration in the trial is for 9 months (till 18 months of age of the child). On week-days, one hen's egg (medium-sized, weighing around 50 g, raw / boiled depending on the mother's choice) will be delivered to children's homes every day by project staff, and mothers will be asked to feed it to the child within four hours. On 5th day, extra raw eggs are supplied to cover for the weekend days. The mothers will be provided instructions on age-appropriate egg-based complementary food recipes through a purpose-made video during the first visit. The importance of feeding the egg to the index child and not sharing it with other family members will also be stressed during monthly counselling sessions to improve adherence to the intervention. This is a model of BCC developed based on evidence generated through best practices for Take –home rations of supplementary nutrition by Government of India.[32] The specific messages were developed through formative research conducted with parents and grandmothers of young children. The project staff will observe the first feeding episode of complementary food containing eggs, and mothers will be counselled in case of minor gastric problems such as cramps in the stomach, nausea and vomiting.

Study staff will visit the households twice a week to observe the child feeding in the first month of the intervention and will encourage and support the mothers. Information on consumption of an egg by the child on the previous day is collected daily by the field staff while delivering the eggs in the intervention group. For comparison, data on eggs consumption will also be collected every month during a home visit in the control group children (Appendix 1).

The eggs will be procured locally from a verified supplier. The quality control measures to ensure food safety of the intervention will include periodic assessment of the microbial contamination of eggs: both at the point of purchase and the point of use. Every month, random sub-samples of eggs collected soon after purchase (3 raw and 3 boiled eggs) and

Confidential: For Review Only

complementary food prepared using eggs for the child (from 6 households every month) will be analysed for microbial contamination (Salmonella, Shigella, total coliform count) at the NIN laboratory. Eggs will be randomly selected for assessing nutrient composition for major nutrients (energy, proteins, iron, folate, calcium, vitamin A, D etc) every three months at the food chemistry laboratory of ICMR- NIN.

Control group

The state government currently provides eggs (16 eggs per month for 7 months to 3 years) for beneficiaries of Integrated child development services (ICDS, a Government initiative for all women and children who can voluntarily enrol) as a part of the Rations. However, during formative research with mothers(n=100), it was found that children below one year of age are not fed whole eggs due to fear of indigestion and other reasons. Moreover, 59% of children were not fed eggs supplied through the government's supplementary nutrition programs due to intra-household sharing.[33] Though there will not be any active intervention by the study team in the control group, all children from both the intervention and control groups may receive eggs as a part of Take-Home Ration, which may reduce the magnitude of the observed effect of the intervention. Also, there is a possibility of e 'contamination' of BCC messages in the control group in this individually randomized trial.

The number of eggs received through Government programme and the number of eggs consumed by the child will be recorded for the intervention as well as control group. We will assess the mother's knowledge of the nutritional benefits of eggs in both the groups at baseline and 18 months for comparison between the two groups (Appendix 1).

Data collection at baseline and during follow up

Data collection in both the intervention and control groups would be done following the same protocol as the observational cohort. The parameters to be measured at different time points are listed in Table 1. Details on the methodology for assessing primary and secondary outcomes are described elsewhere.[25] A trained phlebotomist will draw a 3 ml venous blood sample at both six months and 18 months in children from the intervention group and a sub-sample of 70 children from the control groups. The methods for assessment of blood parameters and epigenetic signatures linked with stunting and

Confidential: For Review Only

cognition have been described elsewhere.[22 25] Data on morbidity (including information on episodes of hospital admission and sick-child clinic visits) will be collected using a structured questionnaire every two weeks. Mothers will be encouraged to report any adverse events to the study staff. Data will be collected using the android interface of CommCare version 2.53 using Samsung A8 tab. The collected data will be securely stored in the database using encrypted codes. For data quality, we have used closed end questions wherever possible, and mandatory fields for core questions. Regex functionality is enabled for anthropometry data. The double data entries are manually managed at the supervisor level.

Withdrawal from the study

During the consent process, mothers will be informed that they may choose to withdraw their child from the study at any time. Children whose mothers wish to withdraw after the randomization will be visited by a project staff to discuss and record the reasons for withdrawal.

In case of suggestive of egg allergy symptoms, the participants would be referred to the PHC, and the mother would be advised to consult the medical officer and seek advice on the continuation of egg supplementation. Data on the prevalence of egg allergy in Indian children is not available but based on the self-reported prevalence of egg allergy (< 2.5%) in other settings,[34] around 1-2 cases are anticipated.

If a participant withdraws from the trial, data captured will contribute to the analysis up to the day prior to discontinuation, unless the mother wishes to withdraw consent for any data captured up to that point. Participants who withdraw from the study will not be replaced.

Statistical analysis

Confidential: For Review Only

We will summarise demographic, clinical, and laboratory data using descriptive statistics. For missing data, multiple imputation method will be used.[35] The primary analysis will be based on the intention to treat (ITT) population and will compare the children's LAZ scores at the age of 18 months in the intervention and control groups using multilevel regression models. We will adjust the models for key covariates (E.g., age, gender and area) associated with the outcome variables. We will check the model assumptions and make appropriate adjustments to the analysis where necessary. We will also use difference-in-difference analysis to evaluate the impact of the intervention. We will compare the secondary outcome measures between the two groups using ITT principles. The study is powered for detecting meaningful differences in linear growth and DHA concentrations in the two groups. Other secondary outcome analyses will be exploratory. Additional analysis of the primary and secondary endpoints will also be presented using the per protocol population. No interim analysis is planned. [36 37]

Patient and Public Involvement

Patients were not directly involved in the design or conduct of the study. Formative research involving mothers, grandmothers and others from the local community (excluding study participants) assisted in understanding local cultural practices and developing BCC material.[33]

Ethics and dissemination

This protocol, informed consent documents, and patient information sheets have been reviewed and approved by the Research Ethics Committees at ICMR-NIN (CR/3/IV/2022 dated 28.7.2022) and LSHTM (IRB no. 18029) as well as by an independent Data Safety Monitoring Board (DSMB). The trial is registered with the Clinical Trials Registry of India (CTRI/2021/11/038208). The DSMB is independent from the sponsor and competing interests and will review clinical data at regular intervals as the study progresses.

Confidential: For Review Only

The study results will be disseminated to the local and national government departments (for potential modifications in the supplementary nutrition program); academic channels like national conferences and published in peer-reviewed scientific journals.

Significance and impact

The objectives of the present trial are well aligned with the objectives of the National Nutrition Mission in India, with the potential for immediate scale-up of the intervention. The study has several strengths. Chief among them is the comprehensive evaluation of outcome indicators ranging from linear growth, cognition, nutritional biomarkers and epigenetic states indicative of exposures and biological function. Additional information on maternal diets and blood biomarkers in pregnancy as well as information on the intrauterine growth of the offspring would allow us to examine pathways for the impact that previous studies have not reported. A limitation is that in this community-based intervention, egg consumption by the index child cannot be supervised completely. Moreover, '''contamination' of the BCC messages in the control group and contamination related to the Government's supplementary nutrition programme may attenuate the effect size. Nevertheless, if the trial demonstrates significant benefits, the evidence on the impact of this relatively inexpensive yet nutrient rich ASF in early life will inform strategies for stunting reduction in India and other developing countries.

Funding

The study is funded by the UK Research and Innovation, Global Challenges Research Fund (EPPHZP5519).

'Authors' contributions:

SKB, SRC, BK: Conceptualized, designed the study and wrote first draft

LA, TD, DP, MC, KS, PA: developed detailed protocols, planned data collection, critical input to manuscript

RM, HD, CH, SFR, RNP, RP, RRK, NKR, MJ, PH: concept, design, critical inputs to manuscript

Confidential: For Review Only

References

1. Hoffman DJ, Sawaya AL, Verreschi I, et al. Why are nutritionally stunted children at increased risk of obesity? Studies of metabolic rate and fat oxidation in shantytown children from Sao Paulo, Brazil. *The American journal of clinical nutrition* 2000;72(3):702-07.
2. Martins VJ, Toledo Florêncio TM, Grillo LP, et al. Long-lasting effects of undernutrition. *International journal of environmental research and public health* 2011;8(6):1817-46.
3. Leroy JL, Frongillo EA. Perspective: What Does Stunting Really Mean? A Critical Review of the Evidence. *Advances in nutrition (Bethesda, Md)* 2019;10(2):196-204. doi: 10.1093/advances/nmy101 [published Online First: 2019/02/26]
4. Comprehensive National Nutrition Survey (CNNS) National Report. Population Council: Ministry of health and family welfare, Government of India, UNICEF, 2018.
5. World Health Organization. Reducing stunting in children: equity considerations for achieving the global targets 2025. Geneva: World Health Organization, 2018.
6. Kulkarni B. Addressing the double burden of malnutrition in developing countries: Need for strategies to improve the lean body mass. *Food and nutrition bulletin* 2018;39(2_suppl):S69-S76.
7. Christian P, Shaikh S, Shamim AA, et al. Effect of fortified complementary food supplementation on child growth in rural Bangladesh: a cluster-randomized trial. *International journal of epidemiology* 2015;44(6):1862-76.
8. Lassi ZS, Das JK, Zahid G, et al. Impact of education and provision of complementary feeding on growth and morbidity in children less than 2 years of age in developing countries: a systematic review. *BMC public health* 2013;13(3):1-10.
9. Radhakrishna K, Hemalatha R, Geddam JB, et al. Effectiveness of zinc supplementation to full term normal infants: a community based double blind, randomized, controlled, clinical trial. *PloS one* 2013;8(5):e61486.
10. Lutter CK, Iannotti LL, Stewart CP. The potential of a simple egg to improve maternal and child nutrition. *Maternal & child nutrition* 2018;14:e12678.
11. Iannotti LL, Lutter CK, Stewart CP, et al. Eggs in early complementary feeding and child growth: a randomized controlled trial. *Pediatrics* 2017;140(1)
12. Stewart CP, Caswell B, Iannotti L, et al. The effect of eggs on early child growth in rural Malawi: the Mazira Project randomized controlled trial. *The American journal of clinical nutrition* 2019;110(4):1026-33.
13. Bierut T, Duckworth L, Grabowsky M, et al. The effect of bovine colostrum/egg supplementation compared with corn/soy flour in young Malawian children: A randomized, controlled clinical trial. *The American Journal of Clinical Nutrition* 2021;113(2):420-27.
14. Keats EC, Das JK, Salam RA, et al. Effective interventions to address maternal and child malnutrition: an update of the evidence. *The Lancet Child & adolescent health* 2021;5(5):367-84. doi: 10.1016/s2352-4642(20)30274-1 [published Online First: 2021/03/11]
15. Papanikolaou Y, Fulgoni VL, 3rd. Eggs Are Cost-Efficient in Delivering Several Shortfall Nutrients in the American Diet: A Cost-Analysis in Children and Adults. *Nutrients* 2020;12(8) doi: 10.3390/nu12082406 [published Online First: 2020/08/17]
16. Iannotti L, Lutter C, Bunn D, et al. Eggs: The uncracked potential for improving maternal and young child nutrition among the world's. *Issue: Nutrition Reviews, 72 (6)* 2014
17. Zeisel SH. Choline, other methyl-donors and epigenetics. *Nutrients* 2017;9(5):445.
18. Tiffon C. The impact of nutrition and environmental epigenetics on human health and disease. *International journal of molecular sciences* 2018;19(11):3425.

Confidential: For Review Only

19. Haggarty P, Ferguson-Smith AC, Kuh D, et al. Life course epigenetics and healthy ageing. *A life course approach to healthy ageing* 2013:198-212.
20. Lorgen-Ritchie M, Murray AD, Staff R, et al. Imprinting methylation predicts hippocampal volumes and hyperintensities and the change with age in later life. *Scientific reports* 2021;11(1):1-12.
21. Lorgen-Ritchie M, Murray AD, Ferguson-Smith AC, et al. Imprinting methylation in SNRPN and MEST1 in adult blood predicts cognitive ability. *Plos one* 2019;14(2):e0211799.
22. Anouschka R, Magatte N, Rajender RaoKalashikam et al. Epigenetic Studies in Families and Children at Risk of Stunting in India, Indonesia, and Senegal. BMJ pediatrics Open, 2022.
23. Kulkarni B, Mamidi R, Balakrishna N, et al. Body composition assessment in infancy and early childhood: comparison of anthropometry with dual-energy X-ray absorptiometry in low-income group children from India. *European journal of clinical nutrition* 2014;68(6):658-63.
24. Department of women and child development. Government of Telangana, 2022.
25. Davies-Kershaw H, Fahmida U, Kyaw HM, et al. Anthropometric, biochemical, dietary, morbidity and wellbeing assessments in women and children in Indonesia, India and Senegal: a UKRI GCRF Action Against Stunting Hub protocol paper. BMJ Pediatrics Open, 2022.
26. Taneja S, Chowdhury R, Dhabhai N, et al. Impact of an integrated nutrition, health, water sanitation and hygiene, psychosocial care and support intervention package delivered during the pre- and peri-conception period and/or during pregnancy and early childhood on linear growth of infants in the first two years of life, birth outcomes and nutritional status of mothers: study protocol of a factorial, individually randomized controlled trial in India. *Trials* 2020;21(1):127. doi: 10.1186/s13063-020-4059-z [published Online First: 2020/02/02]
27. Munoz-Chereau B, Ang L, Dockrell J, et al. Measuring early child development across low and middle-income countries: A systematic review. *Journal of Early Childhood Research* 2021;19(4):443-70.
28. Fernandes M, Villar J, Stein A, et al. INTERGROWTH-21st Project international INTER-NDA standards for child development at 2 years of age: an international prospective population-based study. *BMJ open* 2020;10(6):e035258.
29. Obradović J, Yousafzai AK, Finch JE, et al. Maternal scaffolding and home stimulation: Key mediators of early intervention effects on children's cognitive development. *Developmental psychology* 2016;52(9):1409-21. doi: 10.1037/dev0000182 [published Online First: 2016/08/10]
30. Koshy B, Karthikeyan A, Bose A, et al. Home environment: Short-term trends and predictors in early childhood from an Indian community birth cohort. *Child: care, health and development* 2021;47(3):336-48. doi: 10.1111/cch.12846 [published Online First: 2020/12/31]
31. Vazir S, Engle P, Balakrishna N, et al. Cluster-randomized trial on complementary and responsive feeding education to caregivers found improved dietary intake, growth and development among rural Indian toddlers. *Matern Child Nutr* 2013;9(1):99-117. doi: 10.1111/j.1740-8709.2012.00413.x [published Online First: 2012/05/26]
32. NITI Aayog and World food programme. Take home ration: Good practices across the States/UTs: NITI Aayog, 2022.
33. Thomas N, Kulkarni B, Augustine L, et al. Formative Study to Identify the Determinants, Current Practices and Knowledge of Mothers related to the Egg Consumption of Young Children in Hyderabad, Telangana. Abstract No. OP-2022-0098. 54th Annual

Confidential: For Review Only

conference of Nutrition Society of India. Hyderabad: ICMR-National Institute of Nutrition, Hyderabad, 2022.

34. Muraro A, Halken S, Arshad S, et al. EAACI food allergy and anaphylaxis guidelines. Primary prevention of food allergy. *Allergy* 2014;69(5):590-601.

35. Kang H. The prevention and handling of the missing data. *Korean journal of anesthesiology* 2013;64(5):402-6. doi: 10.4097/kjae.2013.64.5.402 [published Online First: 2013/06/07]

36. McCoy CE. Understanding the Intention-to-treat Principle in Randomized Controlled Trials. *The western journal of emergency medicine* 2017;18(6):1075-78. doi: 10.5811/westjem.2017.8.35985 [published Online First: 2017/11/01]

37. Rosenblum M. Robust Analysis of RCTs Using Generalized Linear Models. Targeted Learning: Springer 2011:187-99.

Confidential: For Review Only

Table 1: Variables measured at different time points

Measure	**Timelines** (intervention and control group)
**Biological sample/ Biomarkers**	
Buccal sample for epigenetics	At birth and at 18 months
**Plasma:** Micronutrient status (Hemoglobin, serum ferritin, serum transferrin receptors, growth hormone, serum iron, zinc, retinol, folate, vitamin B12, folate, IGF1, IGFBP3)	At 3rd trimester of pregnancy and at 6 and 18 months
Blood biomarkers relevant for egg intervention: RBC fatty acids, amino acids, choline, betaine	At 6 and 18 months (in the intervention group and in a sub-sample of 70 children from control group)
Inflammatory markers in blood: CRP and AGP	At 6 and 18 months
**Stool sample:** for entero-pathogens, parasites, and inflammatory markers MPO, AAT	At 6 and 18 months
Micronutrient content of the breastmilk samples: vitamin A, B12, folate	At 3 months postpartum
**Diet, anthropometry and cognition**	
**Diet**<br>● Multiple pass 24-hour diet recall with repeat in 10% of the sample<br>● Initiation of breast feeding<br>● Exclusive breastfeeding and introduction to complementary foods	 Dietary 24-hour recall at 6, 9, 12 and 18 months<br>Breast feeding questionnaire at birth<br>Breastfeeding questionnaire at 1-6 months

Confidential: For Review Only

**Anthropometry measurements**<br>Standing and sitting height, weight, skinfolds and MUAC<br>Length, weight, Head circumference, MUAC, knee-heel length and skinfolds	At 3rd trimester of pregnancy and at 3,6,9,12, 18 and 24 months at birth. Only weight only at 1, 2, 4 and 5 months.
**Assessment of cognition with context specific tests**<br>Home Observation Measurement of the Environment (HOME -Scale)<br>Oxford Neurodevelopment Assessment (OX-NDA)<br>INTER- Neurodevelopment assessment (INTER-NDA):	 At 10-14 months and 18 months<br>At 10-14 months<br>At 22-24 months
**Assessment of morbidity** using structured pictorial charts	Completed by mother every day from birth until 18 months or every two weeks recall by the enumerator throughout the follow up
**Assessment of home environment**	Using a structured questionnaire at 9 months

IGF1: Insulin like growth factor-1; IGFBP3: Insulin like growth factor binding protein 3; CRP: c-reactive protein; AGP: alpha 1-gycoprotein

MUAC: Mid upper arm circumference; MPO: Myeloperoxidase, AAT: alpha-1-antitrypsin

Confidential: For Review Only

Figure 1: CONSORT Flowchart of the study design

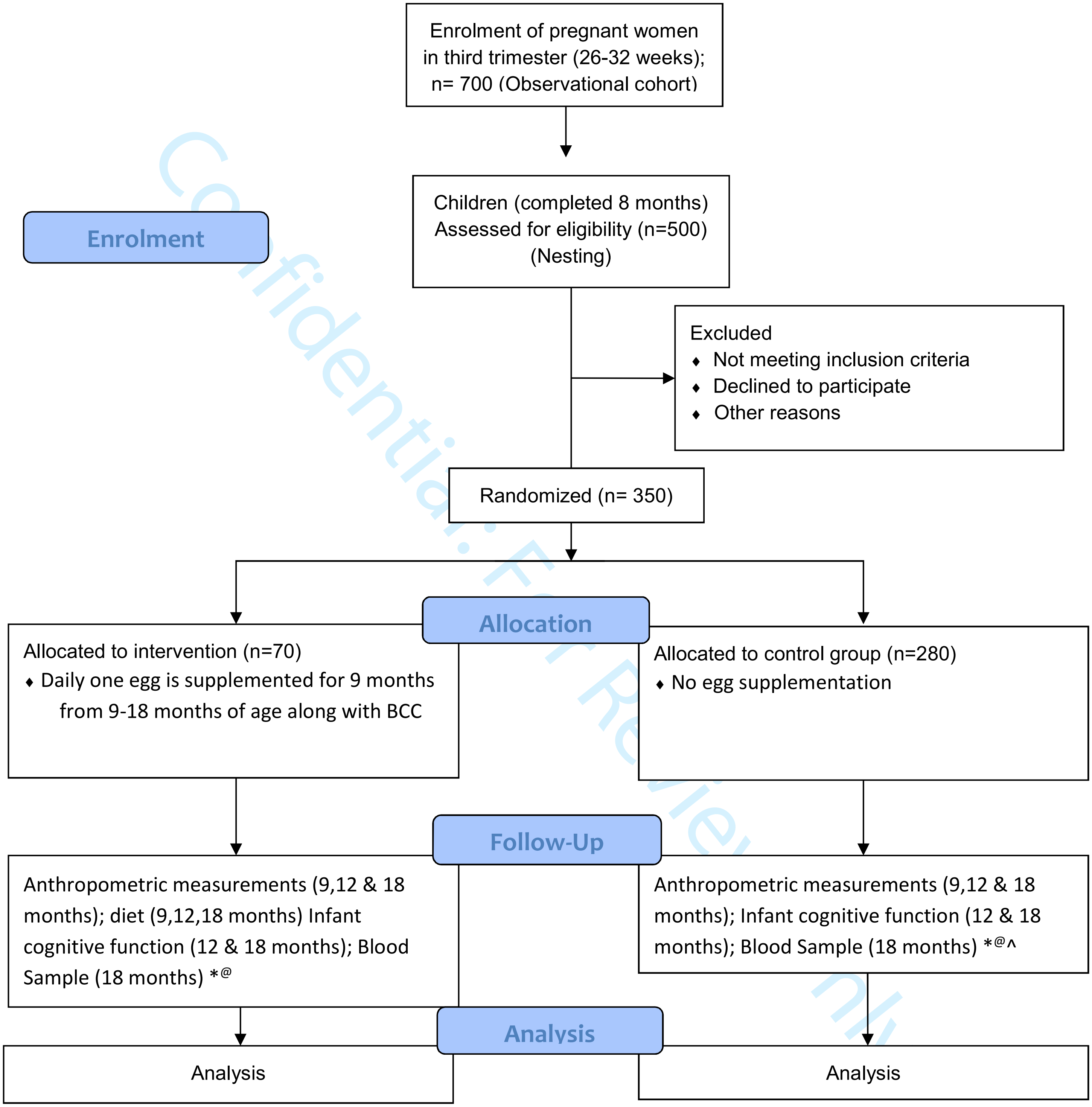

* Blood sample is collected at 6 months as part of observational cohort and at 18 months as part of the nested trial.
\+ additional blood parameters (fatty acids, amino acids, choline, betaine) at 18 months
^ In control group in a subsample of 70 randomly selected children

Confidential: For Review Only

Appendix 1: Questionnaires for data collection

1. Egg preparation and delivery

2) Date and time of the cooking (Date and time)

3) When it was delivered (Time)

4) Did the child receive the egg with in the time (4 h from cooking time)?

a) Yes

b) No

4) If no, what is the reason

1. Traffic

2. The mother/ family members were not available at home

3. The mother/ family members rejected the egg

96. Other specify

2. Daily Compliance questionnaire

1 Did the child receive the egg yesterday

1. Yes

2. No

2 If yes, did the child consume the egg yesterday

1. Yes

2. No

3 Which portion of the egg is given to the child?

1. Only egg white

2. Only egg yolk/yellow

3. Whole egg

4 At what time the child consumed the egg

Date and time (recorded)

6 If No, why was it not consumed yesterday

1. The child disliked the taste

2. The child fell sick/illness/diarrhoea/vomiting

3. Family members/others advised not to give

96. Other, specify

Confidential: For Review Only

3. Monthly follow-up questionnaire (Control)

1. Are you getting eggs from AWC: yes/No
2. If yes how many eggs did you receive last month?
   a. 16 b. >16 c.<16
3. Have you started egg feeding: Yes/No
4. If Yes, how frequently eggs were consumed in the previous week
   a. Everyday b. 4-6 days c. 1-3 days d. Never
5. What is the portion size usually eaten by the child
   a. >1 egg. B. 1 egg. C. ½ egg. D. ¼ egg
6. Was the egg mixed with other food for consumption: Yes/ No
7. Number of times egg eaten per day
   a. Once. B. 2 times. C. 3 times. D. More than 4 times
8. Which portion of the egg was readily accepted by the child:
   a. Yolk. B. White. C. Whole D. None
9. Did your child experience any of the following difficulties after egg consumption
   a. Fever. B. Cough. C. Vomitings& Diarrhoea. D. Wheezing. E. tooth ache F None
10. Have you noticed any change in the child's appetite
    a. Yes, increased. B. Yes, Decreased. C. No change
11. In the previous week have you fed eggs procured from any other sources:yes/No
12. If yes, mention the source___________

Confidential: For Review Only

4. Pre-intervention questionnaire (knowledge and practice), (in intervention and control at endline; only in intervention at the beginning and at midline)

PART 1: PRACTICE

1. Do you currently breastfeed your child?
   a. Yes
   b. No

2. How many times do you breastfeed the child in a day? (Duration of breastfeeding should be at least 10 minutes)
   a. Less than 4 times
   b. 4 times
   c. 5-6 times
   d. More than 6 times

3. Do you give complementary food to your child?   [Dropdown]
   a. Yes
   b. No

4. At what age did you initiate complementary feeding to your child?
   a. Less than 6 months
   b. 6 months
   c. 7 months
   d. 8 months
   e. Not yet started,
   f. Other, specify_________

5. If not yet started, what is the reason for not starting complementary feeding to your child?
   a. Child is too small to eat solid foods
   b. Child doesn't have teeth to chew food
   c. Elders advised not to
   d. Fear of indigestion

6. What are the types of complementary foods that are currently given to the child? [Multiple options can be selected]
   a. Not yet started
   b. Formula milk (Eg: Lactogen,amul milk spray etc . . .)
   c. Commercial baby foods (Eg: Ceralac, farex,nestem etc. . .)
   d. Homemade semi-solids (Eg: Uggu, rice+ dhal (soft cooked), mash fruits&vegetables etc. . .)
   e. Processed food (Eg:Biscuits,kurkure,chips etc . . .)

Confidential: For Review Only

f. Homemade solids (Eg: Normal food)
g. Other, specify_______

7. What are the foods that are generally included in the homemade complementary foods? (logic)[Multiple options can be selected]
a. Cereals & millets
b. Pulses
c. Green leafy vegetables
d. Roots & tubers
e. Other vegetables
f. Fruits
g. Flesh foods
h. Eggs
i. Milk & milk products
j. Fat & oils
k. Sugar & jaggery.

8. Do you feed eggs to your child? Skip Logic (Questions regarding feeding egg, only if the mother is feeding egg to her child)
a. Yes
b. No

9. If yes, at what age did you start feeding eggs to your child?
a. 6 months
b. 7 months
c. 8 months
d. 9 months
e. Other, specify _________

10. If not, what is the reason for not initiating egg to the child before 9 months?
a. The eggs do not get digested
b. The eggs cause heat build-up in the child's body
c. Family members suggested to avoid eggs till 8th month
d. Eggs are high in fat/cholesterol
e. Other, specify_________________

11. Who suggested not to initiate egg to your child?
a. Husband
b. Mother-in-law
c. Neighbours
d. Doctor
e. Other, specify ____________

12. What form of the egg did your child like to eat?
a. Boiled Egg
b. Half boiled
c. Omelette /Scrambled Egg
d. Egg Curry
e. Mashed with rice
f. Other, specify ____________

Confidential: For Review Only

13. Did you stop feeding eggs to your child after initiating?
    a. Yes
    b. No

14. If yes, why did you stop feeding eggs to your child?
    a. The child disliked the taste
    b. The child had an allergy
    c. The child had diarrhoea/vomiting
    d. Family members/others advised not to give.
    e. Other, specify___________________

15. If the child did not like the taste, what did you do?
    a. Tried a different recipe with egg
    b. Stopped feeding egg
    c. Other, specify_____________

16. If the child developed an allergy/diarrhoea/ vomiting after consuming the egg, whom did you contact?
    a. Government hospital / PHC doctor
    b. Private doctor/Registered Medical Practitioner
    c. Local medical shop
    d. Traditional/ Religious leaders
    e. Treated at home
    f. Family members/neighbours suggested
    g. Other, specify _____________

17. Does any member of the family have an allergy to any food items?
    a. Yes
    b. No

18. Who has an allergy to the food item?
    a. Father of the child
    b. Mother of the child
    c. Grandparents of the child
    d. Siblings

19. Which food is allergic to the above-mentioned person?
    ____

20. How do you feed eggs to your child? [MCQ- Multiple options can be selected]
    a. Mashed with rice
    b. Boiled Egg
    c. Half boiled
    d. Omelette /Scrambled Egg
    e. Egg Curry
    f. Other, specify __________________

21. Which part of the egg is given to the child? [Drop down]

Confidential: For Review Only

a. Only egg white
b. Only egg yolk
c. Whole egg

22. Why do you give only the white part of the egg to the child? [If selected a]-
a. Child likes the taste
b. Easy to swallow
c. Family members / elders advised
d. Doctor advised
e. ASHA/ ANM/Anganwadi worker advised.
f. Other, specify_________________

23. Why do you give only the yellow part to the child? [If selected b]
a. Child likes the taste
b. Easy to swallow
c. Family members/elders advised
d. Doctor advised
e. ASHA/ ANM/Anganwadi worker advised.
f. Other, specify _________________

24. How frequently do you feed eggs to your child? [Drop down]
a. Daily
b. Twice a week
c. Thrice a week
d. >4 times a week
e. Weekly once

25. Usually, how many eggs do you feed your child in a day? [MCQ]
a. One whole egg
b. Half egg
c. Two whole eggs
d. One whole egg white
e. Two whole egg white
f. One whole egg yolk
g. Two whole egg yolk
h. Other, specify___________________

26. If the egg is not fed -, please tell us the reason from the options provided below [Multiple options can be selected]
a. Not available and accessible
b. The child dislikes eating egg
c. No time to cook the egg
d. Family members do not prefer
e. Other, specify__________________

27. Do you avoid feeding eggs to your child on festivals/special days in a week? [Skip Logic-If yes, go to Q. 21, If not, go to 23]
a. Yes
b. No

28. If yes, please mention on which days in the week do you avoid feeding eggs to the child? [Short answer]

Confidential: For Review Only

____

29. If yes, please mention on which occasions do you avoid feeding eggs to the child? (Prompt: Festivals, seasons) [Short answer]

____

Confidential: For Review Only

PART 2: KNOWLEDGE

This section is to know the knowledge of the mother regarding feeding egg to the child

30. Are eggs an important component of the child's diet for their growth and development [Drop down]
    a. Yes
    b. No
    c. Don't know

31. What are the benefits of feeding an egg to the child? [Multiple options can be selected]
    a. Eggs are good for vision
    b. Eggs help the child grow tall
    c. Eggs provide immunity
    d. Eggs are good for bones and teeth
    e. Eggs increase brain capacity
    f. All of the above
    g. None of the above
    h. I don't know
    i. Other, specify________

32. Which part of the egg is healthy for the child? [Drop down]
    a. Only Egg yolk
    b. Only Egg white
    c. Whole egg
    d. Don't know

33. At what age the children should start consuming eggs? [Drop down]

    ____

34. I intend to feed one whole egg daily to the child from 9 months of age [Drop down]
    a. Extremely unlikely
    b. Unlikely
    c. Neutral
    d. Likely
    e. Extremely likely

35. I am confident that I can feed one whole egg to the child every day from 9 months of age [Drop down]
    a. Not confident
    b. Confident
    c. Very confident
    d. Neutral

36. Husband and elders in the family will support in feeding egg to the child from 9 monthsof age [Drop down]

Confidential: For Review Only

a. Agree
b. Disagree
c. Don't know

37. Would you like to know about the recipes you can prepare and feed your child? [Dropdown]
a. Yes
b. No

ICDS

38. Did you enrol your or your child's name in the local ICDS center/ Anganwadi? [SkipLogic- If not, go to 44]
a. Yes
b. No

38. a If yes, What are the benefits you are getting from the ICDS centre (Multiple choices can be selected)
a. Arogya Lakshmi one full meal (During pregnancy and lactation of 6 months)
b. Balamrutham
c. Eggs
d. Iron and folic tablets
e. Milk
f. Others, please specify____________________________

39. How many eggs do you receive from the ICDS center in a month?
a. 16
b. Less than 16
c. Don't know /remember

40. Have you received less than 16 eggs anytime from the ICDS center/ Anganwadi?
a. Yes
b. No

41. If yes, do you know the reason for receiving less than 16 eggs from the ICDS center?
a. No
b. Yes, specify

42. How frequently are the eggs given from the ICDS center are consumed by your child in the last 30 days? [Skip Logic-Skip next question, if selected Option. a, Go to next question if selected option. b./c./d.]
a. Often
b. Rarely
c. Never consumed

43. If the number of eggs fed is less than the allowance, what is the reason? [MCQ]
a. Not received or received less number of eggs from the center
b. The child is too young to eat the egg
c. Shared it with family members
d. Discarded
e. Other, specify ______

Confidential: For Review Only

44. Is there any organization/non-governmental organization (NGO) that sponsors eggs for your family?
    a. Yes
    b. No

45. If yes, what is the name of the organization? (Short answer)

____

46. What is the number of eggs that you receive from the organization mentioned above?

____

5. Preliminary screening (For both intervention and control)

1. Will you relocate to a new place in the next 9 months period?

a.Yes

b.No

c.Maybe

2.Do you give complementary food to your child?

a.Yes

b.No

3.Do you consume eggs at your home?

a. Yes

b.No

4.Have you started feeding eggs to your child?

a. Yes

b.No

5.Does your child have allergic reactions after consumption of egg?

a.Yes

b.No

c. Don’t know

6.Have any other allergic reactions to any other food item occurred to your child?

Confidential: For Review Only

a. Yes

b. No

c. Don’t know

7. Which food item is your child allergic to?

____

8. Is any of the family member allergic to any food items?

a. Yes

b. No

c. Maybe

9. Which family member has allergic reactions to food item?

a. Mother b. Father c. Siblings

d. Grandparents.

10. Which food item does the above-mentioned person has allergic reactions to?

____

11. Does your child have any of the below mentioned conditions?

a. Congenital anomalies/diseases

b. Chronic morbidity

c. None

12. Will you be willing to initiate egg consumption for your child and be a part of this egg supplementation group if randomly selected from 9 to 18 months of your child's age?

a. Yes

b. No

13. Did your child get MMR vaccination?

a. Yes

b. No

Confidential: For Review Only

Appendix 2

Protocol for Management of egg allergy

Since egg allergies typically present immediately or within two hours after ingestion, an auxiliary nurse midwife will be present during the first egg feed and she will observe the child for any for signs of egg allergies or anaphylaxis for a period of 2 hours. In case of allergy/anaphylaxis, allergies will be clinically evaluated by a paediatrician and treated clinically with a referral to tertiary hospital for management after giving a dose of antihistamine in the field unit of the study.

Second generation antihistamines and the dose will be kept with the Staff nurse for use in case of emergency.

-Cetirizine 6 months to < 2 years: 0.25 mg/kg;

- Levocetirizine 6 months to 5 years: 1.25 mg OD

Any child exhibiting egg allergy will be excluded from the study.

Confidential: For Review Only
